# Emergence of large-scale patterns in soft quasicrystals

**DOI:** 10.1038/s41467-026-71816-y

**Published:** 2026-04-22

**Authors:** Dean Chen, Nitesh Arora, Yuhai Xiang, Qi Yao, Quan Zhang, Stephan Rudykh

**Affiliations:** 1 Department of Mechanical and Aerospace Engineering, University of California, Los Angeles, Los Angeles, CA USA; 2 School of Chemical and Biomolecular Engineering, Georgia Institute of Technology, Atlanta, GA USA; 3 Department of Mechanical Engineering, Indian Institute of Technology Jodhpur, Rajasthan, India; 4 Tokyo Electron America Inc, Austin, TX USA; 5 Department of Mechanical Engineering, University of Wisconsin-Madison, Madison, WI USA; 6 School of Mathematical and Statistical Sciences, University of Galway, Galway, Ireland; 7 Cavendish Laboratory, Department of Physics, University of Cambridge, Cambridge, UK

**Keywords:** Polymers, Composites

## Abstract

We report the experimental observations of large-scale pattern formation in soft quasicrystals activated by mechanical loading. The soft material system utilizes localized transformations to give rise to large-scale organized patterns. The complex multiscale mechanisms lead to an initial loss of microscale quasicrystalline order, which ultimately reemerges in the large-scale patterns with rotational symmetry. Our experiments demonstrate that the resulting large-scale patterns can be pre-designed by fine-tuning initial local chirality and porosity at small length scales. Furthermore, different initial configurations result in patterns exhibiting high shape similarity, with characteristic lengths falling within a discrete sequence governed by the silver ratio. This experimentally observed phenomenon opens a new class of transformative materials with switchable self-similarity propagating across length scales.

## Abstract (web summary)

The study of quasicrystals has revealed unique material properties that challenge traditional notions of order and symmetry in solids, prompting interest in their potential applications in advanced materials. Here, authors demonstrate the experimental realization of large-scale pattern formation in soft quasicrystals through mechanical loading, revealing a multiscale transformation process that can be finely tuned by adjusting initial local chirality and porosity.

## Introduction

The realm of metamaterial design has been dominated by the periodic structure concept^1–7^. However, there is an intriguing potential for discovering unusual material behavior outside the limits of periodicity. Aperiodic patterns—from the intuitive designs of ancient architectural ornaments to the rigor of modern mathematics^8,9^—have long intrigued humanity through their complexity, representing a sophisticated class of order capable of unlocking unprecedented material behaviors. The epochal discovery of quasicrystals^10,11^ presented a paradigm shift in our understanding of ordered solids, challenging their exclusive association with periodicity. Unlike their periodic counterparts, a quasicrystalline pattern can continuously fill the space with a long-range order without having translational symmetry. This unique characteristic allows materials to exhibit symmetries that are forbidden in periodic crystals, such as the 5-^10^, 8-^12^, 10-^13^, and 12-fold^14–17^ rotational symmetry, leading to unique physical properties such as novel magnetic^18^ and photonic^19^ phenomena, distinctive thermal and conductive behaviors^18,20^, and chiral acoustic phonons^21^, with more yet to be discovered.

Given the extraordinary potential of quasicrystals, an intriguing question emerges: can these unusual material structures be actively triggered or further manipulated using mechanical forces in soft matter? As we shall show, in coarse-grained, macroscopically homogeneous porous materials, quasiperiodic patterns can break locally and reemerge at larger length scales, through deformation-activated, multiscale mechanisms. To achieve this, we introduce finely tuned chirality into the void geometry to encode nucleation seeds and reconstruct quasicrystalline order through convergent propagation of local microstructural transformations. Our computational exploration identifies the specific chiral configurations that give rise to large-scale pattern formations, which are subsequently realized through experiments. Notably, different initial configurations produce large-scale patterns across diverse length scales while maintaining high morphological similarity. Intriguingly, these characteristic lengths are not arbitrary but conform to a discrete sequence governed by the silver ratio.

## Results

### Experimental results

**Fig. 1:**
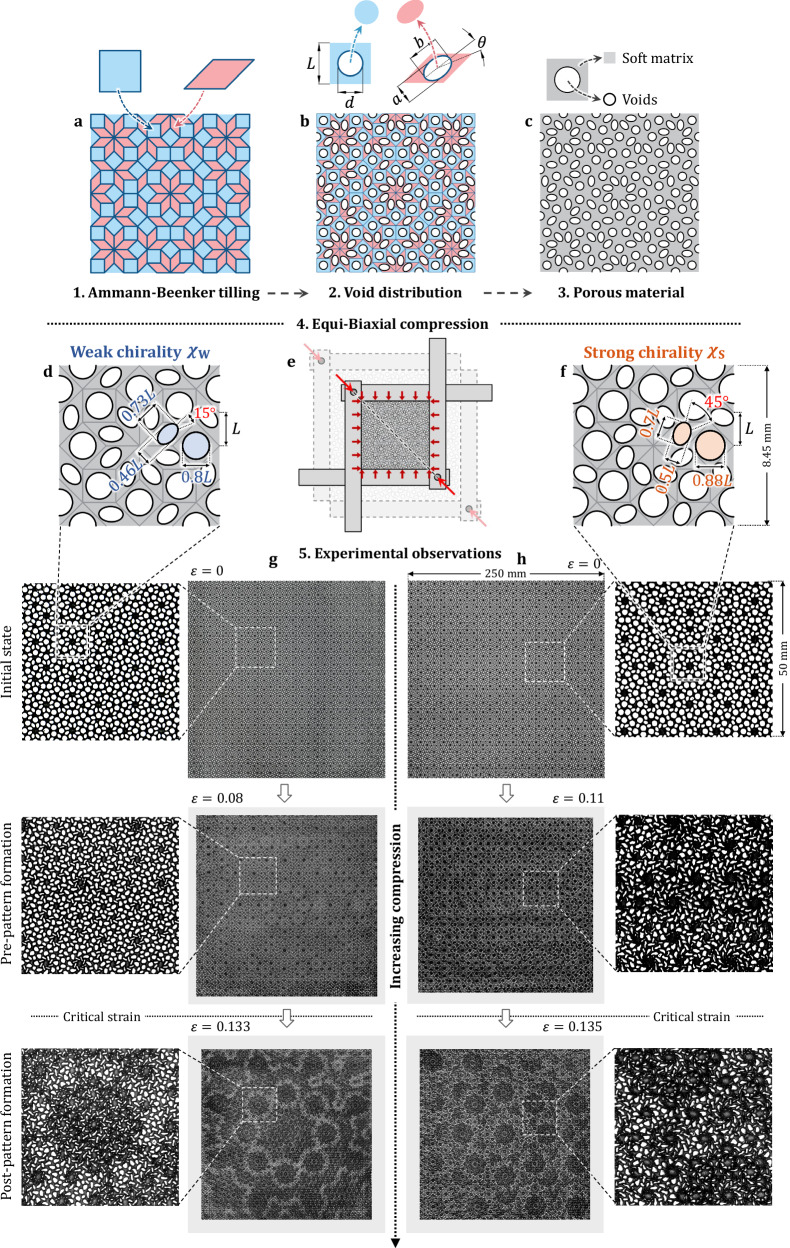
Experimental observation of large-scale pattern formation in soft quasiperiodic materials. **a** Ammann-Beenker quasiperiodic tiling with square and $45^\circ$-$135^\circ$- rhombic prototiles. **b** Void distribution in Ammann-Beenker base tiling. **c** Quasiperiodic porous microstructure. **d** Initial geometry of weak chirality (${\chi }_{{{{\rm{W}}}}}$) material sample. **e** Experimental setup for equi-biaxial plane-strain compression. **f** Initial geometry of a strong chirality (${\chi }_{{{{\rm{S}}}}}$) material sample. **g** Experimental observations of large-scale pattern formation in the weak chirality (${\chi }_{{{{\rm{W}}}}}$) sample under strain levels $\varepsilon=0$, $0.08$, and $0.133$. **h** Experimental observations of large-scale pattern formation in the strong chirality (${\chi }_{{{{\rm{S}}}}}$) sample under strain levels $\varepsilon=0$, $0.11$, and $0.135$.

The specimens are fabricated as a square porous sheet, with voids embedded in a soft elastomeric matrix and arranged in a quasicrystalline configuration (Fig. 1c). Specifically, the distribution of voids follows an eightfold Ammann-Beenker quasiperiodic tiling pattern^12,22^ comprising square and $45^\circ$-$135^\circ$ rhombic prototiles, as shown in Fig. 1a. The voids are centered within each prototile: a circular void (of diameter $d$) at the center of each square tile, and an elliptical void (with major diameter $b$ and minor diameter $a$) at each rhombic tile’s centroid (see Fig. 1b). The elliptical voids also feature a counter-clockwise chirality rotation, $\theta$, relative to the rhombus’s major axis. The quasiperiodic porous material sheets, incorporating circular and elliptical voids, are directly fabricated through 3D printing of soft polymer materials (specifically, Stratasys Agilus30 FLX935 mixed with Vero Black Plus RGD875 as FLX9860-DM).

Large-scale pattern formation was observed only for specific material configurations, identified through a broader experimental sweep of the geometric parameters. We highlight two successful realizations of these patterns: one featuring lower chirality, denoted as ${\chi }_{{{{\rm{W}}}}}$ (weak chirality), and another with higher chirality, denoted as ${\chi }_{{{{\rm{S}}}}}$ (strong chirality). The specific initial geometries for these samples are: I) $b/L=0.73$, $a/L=0.46$, $d/L=0.8$, and $\theta=15^\circ$ (as shown in Fig. 1d) and II) $b/L=0.7$, $a/L=0.5$, $d/L=0.88$, and $\theta=45^\circ$ (as shown in Fig. 1f), where $L$ represents the characteristic side length of both the square and rhombic prototiles. The two samples are characterized not only by their initial chiralities but also by their initial porosities, which are globally averaged as ${\bar{\rho }}_{0}^{{{{\rm{W}}}}}\approx 0.438$ and ${\bar{\rho }}_{0}^{{{{\rm{S}}}}}\approx 0.498$, respectively. In our experimental tests, both samples possess a width of ${W}_{{{{\rm{S}}}}}=250{{{\rm{mm}}}}$ and a thickness of $10\,{{{\rm{mm}}}}$; the actual prototile length $L$ in the printed sample is $L=1.75{{{\rm{mm}}}}$. In our experiment, the equi-biaxial compression was applied to the samples as shown schematically in Fig. 1e. A constant slow strain rate of $2.5\times {10}^{-3}{{{{\rm{s}}}}}^{-1}$ was used to minimize the viscoelastic behavior of the soft material.

Figure 1g, h shows the experimental observations of large-scale pattern formation in the weak (${\chi }_{{{{\rm{W}}}}}$) and strong (${\chi }_{{{{\rm{S}}}}}$) chirality samples, respectively. Each column of photos corresponds to the same material sample subjected to different strain levels of equi-biaxial compression. In each photo, the bright areas correspond to voids, whereas the dark areas correspond to the matrix material. Thus, the higher porosity areas are brighter, while the lower porosity areas are represented by the darker domains.

In the undeformed state ($\varepsilon=0$), the microstructures are initially characterized by a macroscopically homogeneous porosity distribution (see Fig. 1g, h). Upon compression, in the weak chirality ${\chi }_{{{{\rm{W}}}}}$ sample ($\theta=15^\circ$), both circular and elliptical voids progressively shrink, leading to an overall reduction in material porosity. At low compressive strain levels (for example, $\varepsilon=0.08$ in Fig. 1g), the porosity distribution remains coarse-grained uniform across the sample. However, upon reaching a critical strain level (approximately $\varepsilon=0.1$), a sudden collapse of voids is observed locally, breaking the material’s initial quasicrystalline organization. Interestingly, collapse does not occur uniformly across the sample; instead, it initiates at discrete locations, which we hereafter refer to as “nucleation spots.” Once nucleated, the local transformations start to propagate outward through successive void collapses along traveling fronts (see Supplementary Movie 1). The void closures along propagation paths manifest in a significant reduction in local porosity, transforming the initially porous structures into highly densified domains. Remarkably, with continued compression, collapse propagation does not densify the entire specimen into a uniformly dark domain but instead becomes progressively confined, leaving narrow paths along which a substantial fraction of voids remains open. For example, at a sufficiently high compressive strain ($\varepsilon \approx 0.133$ in Fig. 1g and Supplementary Data 1), we still observe large porous regions that remain clearly delineated from the surrounding dark, densified domains. Hereafter, we refer to these confined dark regions (where the backlight is predominantly blocked due to void closure) as “densified domains,” and the bright, stripe-like paths with sufficient porosity as “high-porosity domains.” Intriguingly, those stripe-like paths reorganize into a large-scale pattern with quasiperiodic order and eightfold symmetry, exhibiting a characteristic length that is significantly larger than that of the original prototiles. Finally, the structure returns to its undeformed configuration upon full release of the macroscopic load, with no noticeable hysteresis during temporary holding at the peak strain or after unloading.

A similar pattern formation trajectory is observed in the material with stronger chirality ($\theta=45^\circ$). Prior to the critical strain, the strong chirality (${\chi }_{{{{\rm{S}}}}}$) sample likewise maintains a coarse-grained porosity distribution. Beyond a critical strain of approximately $\varepsilon \approx 0.11$ (Fig. 1h), nucleation initiates at locations similar to those observed in the ${\chi }_{{{{\rm{W}}}}}$, but the subsequent propagation follows distinct paths. As voids progressively close along these traveling fronts, the propagation of transformation gradually approaches a steady state and becomes confined to specific domains, while preserving large porous regions at a sufficiently high strain ($\varepsilon \approx 0.135$, Fig. 1h and Supplementary Data 2). Notably, in the ${\chi }_{{{{\rm{S}}}}}$ sample, the propagation of transformation is confined to smaller domains and does not extend as far as in ${\chi }_{{{{\rm{W}}}}}$ sample. Eventually, the ${\chi }_{{{{\rm{S}}}}}$ sample forms a noticeably different pattern at $\varepsilon \approx 0.135$ (Fig. 1h) compared to that in the ${\chi }_{{{{\rm{W}}}}}$ sample at $\varepsilon \approx 0.133$ (Fig. 1g). Nevertheless, we find that the ${\chi }_{{{{\rm{W}}}}}$ and ${\chi }_{{{{\rm{S}}}}}$ samples exhibit closely related pattern morphologies, as the pattern formed in the ${\chi }_{{{{\rm{W}}}}}$ sample appears as a magnified counterpart of that in the ${\chi }_{{{{\rm{S}}}}}$ sample. This morphological similarity is demonstrated more clearly through numerical results presented later. Since both samples share the same prototile size in their initial geometries, this observation indicates that large-scale pattern formation in quasiperiodic porous materials can be systematically tuned across a broad range of characteristic lengths.

In this section, we experimentally demonstrate large-scale pattern formation in quasiperiodic porous materials. Notably, the two successful cases (${\chi }_{{{{\rm{W}}}}}$ and ${\chi }_{{{{\rm{S}}}}}$ samples) are rare outcomes among the broad parameter sweep conducted in this study, in which most tested configurations fail to develop large-scale patterns. These unsuccessful configurations instead evolve into one of two distinct failure-to-form modes (Supplementary Results 1): mode A (“failed nucleation”), characterized by nearly simultaneous collapse across the entire sample without identifiable nucleation or pattern formation (Supplementary Fig. 1a), or mode B (“trapped nucleation”), in which nucleation initiates but is arrested at small length scales, preventing the emergence of large-scale patterns (Supplementary Fig. 1b). Overall, among the successful cases, we consistently observe that the formation of large-scale patterns proceeds through a common multiscale, three-stage process: (i) localized collapse nucleates at discrete locations at small length scales, (ii) the transformation spreads outward from these nascent nuclei across increasing length scales, and (iii) ultimately converges within well-defined boundaries at large length scales.

### Numerical results

To obtain detailed insights into a deeper understanding of the multiscale pattern formation, we perform numerical simulations. Specifically, we conduct large-deformation finite element analyses using the solid mechanics module in COMSOL Multiphysics 6.0 (the computational implementation is detailed in the Methods section). This section first presents the numerical results for the ${\chi }_{{{{\rm{W}}}}}$ and ${\chi }_{{{{\rm{S}}}}}$ samples under loading conditions corresponding to those applied in the experiments, and then extends the analysis to a broader parameter space to elucidate how the initial chirality and porosity govern large-scale pattern formation.

**Fig. 2:**
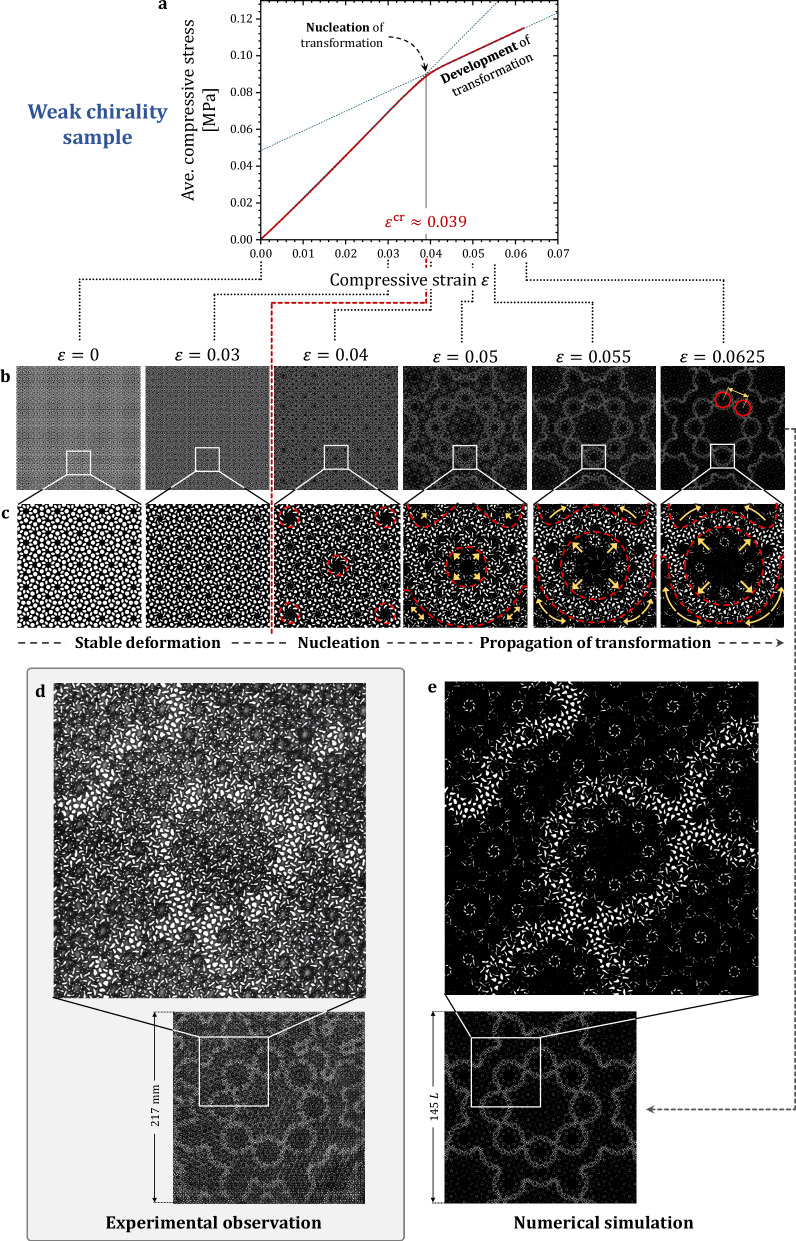
Numerical results of large-scale pattern formation in the weak chirality (χw) sample. **a** Average compressive stress as a function of equi-biaxial strain for ${\chi }_{{{{\rm{W}}}}}$ sample. **b** Material sample from prior- to post-transformation states corresponding to strain levels $\varepsilon=0$, $0.03$, $0.04$, $0.05$, $0.055$, and $0.0625$ (deformation visually amplified by 3). **c** Zoom-in images of material samples. Comparison of post-transformation patterns: numerical (**e**) vs experimental (**d**) results for strain level $\varepsilon \,=\,0.0625$ (simulations) and$\,0.133$ (experiments). Source data are provided as a Source Data file.

Figure 2a shows the average compressive stress as a function of compressive strain $\varepsilon$ for the ${\chi }_{{{{\rm{W}}}}}$ sample. Below the curve, in Fig. 2b, corresponding material patterns are displayed for various strain levels (see also the animation in Supplementary Movie 2). For visualization purposes, the displayed displacement field is uniformly amplified by a factor of 3, without affecting the underlying deformation, strain, or stress fields in simulations. At low enough compression levels, the stress-strain curve exhibits nearly linear behavior (as seen from the red curve of Fig. 2a for strain levels below ${\varepsilon }^{{{{\rm{cr}}}}}\approx 0.039$). Only slight local densification is observed across the sample; nevertheless, the material retains a coarse-grained homogeneous porosity, and no nucleation is initiated. However, upon reaching a critical strain ${\varepsilon }^{{{{\rm{cr}}}}}\approx 0.039$, the material enters a rapid softening regime, manifesting in the curve slope decrease from $d\sigma /d\varepsilon \,\approx 2.29\,{{{\rm{MPa}}}}$ to $1.09\,{{{\rm{MPa}}}}$. However, within this softening regime, the slope change does not correspond to a sharp turning but instead occurs as a rapid but smooth transition. This behavior coincides with collapse initiating at discrete locations (nucleation spots), rather than occurring uniformly across the entire sample. We observe that, around these nucleation spots, the array of chiral elliptical holes is already softened by inward contraction at early stages prior to the critical strain, as evidenced at $\varepsilon=0.03$ in Fig. 2b. This early-stage softening does not significantly affect the global mechanical response, but creates localized sites that are prone to instability, hereafter referred to as “nucleation seeds.”

Once nucleated, the microstructural transformations begin to propagate with further compression, and densified domains are observed to grow. Interestingly, the growth of the “densified domains” differs based on their nucleation positions. To illustrate this microscale process, we show a zoom-in at the microstructure level of ${\chi }_{{{{\rm{W}}}}}$ sample in Fig. 2c. As shown in Fig. 2c at $\varepsilon=0.05$, $0.055,$ and $0.0625$, nucleation initiated at the center of the image propagates outward continuously, without interference from transformations nucleated elsewhere, ultimately forming complete eightfold domains. The resulting eightfold subdomains, marked by red octagons in Fig. 2b at $\varepsilon=0.0625$, appear as paired counterparts that define the pattern shape and are referred to as “characteristic domains,” as detailed further in the Large-scale characterization section. In contrast, densified domains nucleated near the outer corners of the image in Fig. 2c propagate while merging with each other, giving rise to more intricate domain morphologies. Eventually, the propagation of microstructural transformation is confined to the specific domains, outlined by red dashed curves in Fig. 2c, at $\varepsilon=0.0625$. We shall demonstrate later in the Large-scale characterization section that the confinement of propagation is a volumetric complementarity arising from the incompressibility of the matrix material.

We compare the large-scale patterns obtained from experiments and numerical simulations, finding good agreement between them (Fig. 2d and Fig. 2e). We also observe that the critical strain identified in experiments ($\varepsilon \approx 0.08$) is larger than that obtained in simulations ($\varepsilon \approx 0.039$). This shift arises from experimental deviations from idealized conditions, including residual out-of-plane deformation under nominal plane-strain clamping, frictional effects at the fixture interfaces, and weak rate-dependent behavior of the polymer, all of which may delay the onset of localized nucleation relative to the ideal numerical model.

**Fig. 3:**
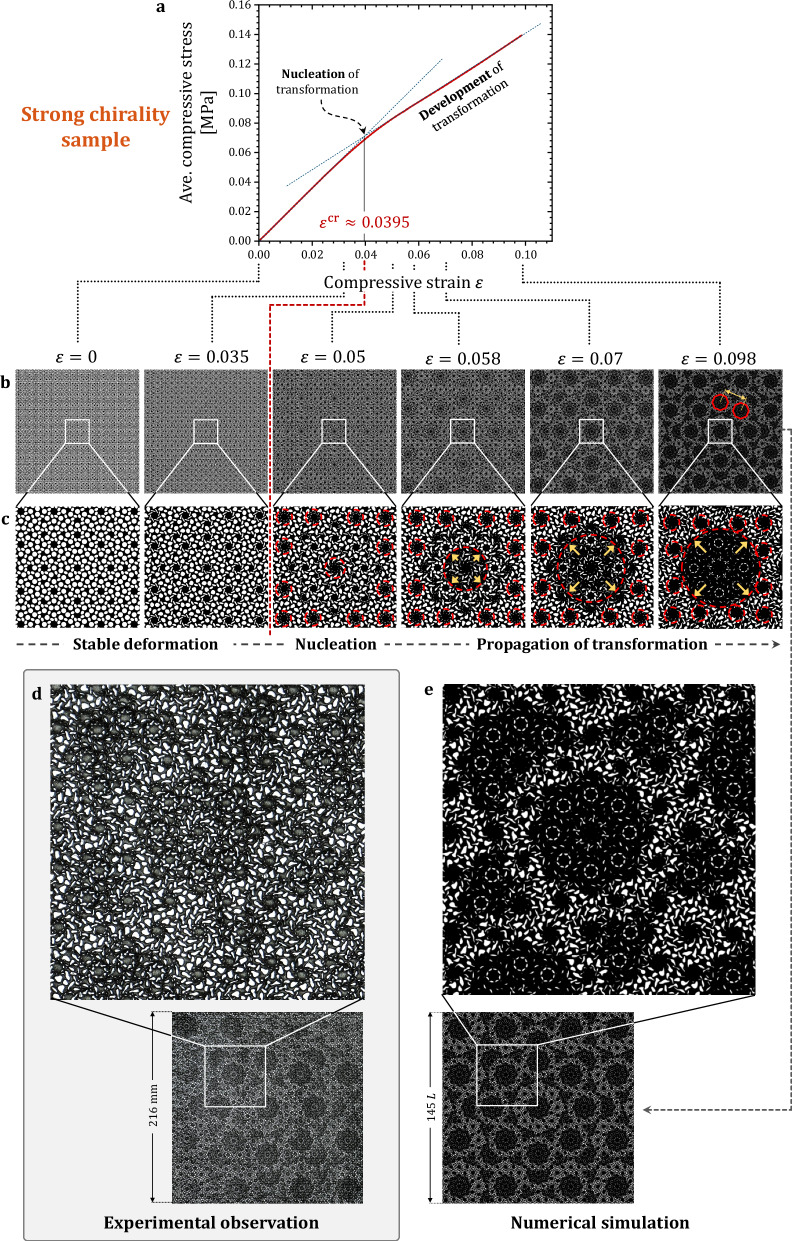
Numerical results of large-scale pattern formation in the strong chirality (χs) sample. **a** Average compressive stress as a function of equi-biaxial strain for ${\chi }_{{{{\rm{S}}}}}$ sample. **b** Material sample from prior- to post-transformation states corresponding to strain levels $\varepsilon=0$, $0.035$, $0.05$, $0.058$, $0.07$, and $0.098$ (deformation visually amplified by 2). **c** Zoom-in images of material samples. Comparison of post-transformation patterns: numerical (**e**) vs experimental (**d**) results for strain level $\varepsilon=0.098$ (simulations) and$\,0.135$ (experiments). Source data are provided as a Source Data file.

The strong chirality (${\chi }_{{{{\rm{S}}}}}$) sample exhibits a similar stress-strain response to the ${\chi }_{{{{\rm{W}}}}}$ sample, with global softening occurring after a critical strain of ${\varepsilon }^{{{{\rm{cr}}}}}\approx 0.0395$ (Fig. 3a). However, the ${\chi }_{{{{\rm{S}}}}}$ sample demonstrates a smaller and smoother slope change, from $d\sigma /d\varepsilon \,\approx 1.78{{{\rm{MPa}}}}$ to $1.12{{{\rm{MPa}}}}$, compared to the ${\chi }_{{{{\rm{W}}}}}$ sample (see also the animation in Supplementary Movie 3). Despite different initial stiffnesses, the two microstructures encounter global softening at very close critical strains (${\chi }_{{{{\rm{S}}}}}$: $0.0395$ and ${\chi }_{{{{\rm{W}}}}}$: $0.039$, approximately). Nevertheless, the softening rate in the strong chirality sample ($37.1\%$ slope decrease) is significantly smaller than that of the weak chirality sample ($52.4\%$ slope decrease); this is consistent with the observation of less extensive propagation of collapse in the ${\chi }_{{{{\rm{S}}}}}$ sample during transformation (Fig. 2e and Fig. 3e). Early-stage softening around chiral elliptical holes is also observed in the ${\chi }_{{{{\rm{S}}}}}$ sample, and it is more pronounced due to the sample’s higher chirality, as seen in Fig. 3c at $\varepsilon=0.035$. However, we also notice that only a few of these early-softened seeds subsequently develop into active nucleation sites upon further compression; most remain trapped sites until the final pattern is established (Fig. 3c at $\varepsilon=0.098$). The subsequent propagation of the transformation also differs in the ${\chi }_{{{{\rm{S}}}}}$ sample. As seen from the comparison between Fig. 2b and Fig. 3b, the trapped nucleation seeds (dark holes in Fig. 3c at $\varepsilon=0.07$) block the merging of densified domains propagated from different nucleation spots, thereby forming more isolated eightfold dark domains (characteristic domains), marked by red octagons at $\varepsilon=0.098$ in Fig. 3b. In contrast, the ${\chi }_{{{{\rm{W}}}}}$ sample develops more extensively merged densified (dark) domains, resulting in the formation of fewer characteristic domains (eight observed in Fig. 2b) than in the ${\chi }_{{{{\rm{S}}}}}$ sample (twenty observed in Fig. 3b).

Despite the distinct distributions of dark (densified) domains, the high-porosity, bright stripes formed in ${\chi }_{{{{\rm{S}}}}}$ and ${\chi }_{{{{\rm{W}}}}}$ samples exhibit strong similarity in shape (Fig. 2e and Fig. 3e). This correlation between the large-scale patterns arising from different chiral microstructures can be quantitatively analyzed, as will be further detailed in the following section.

**Fig. 4:**
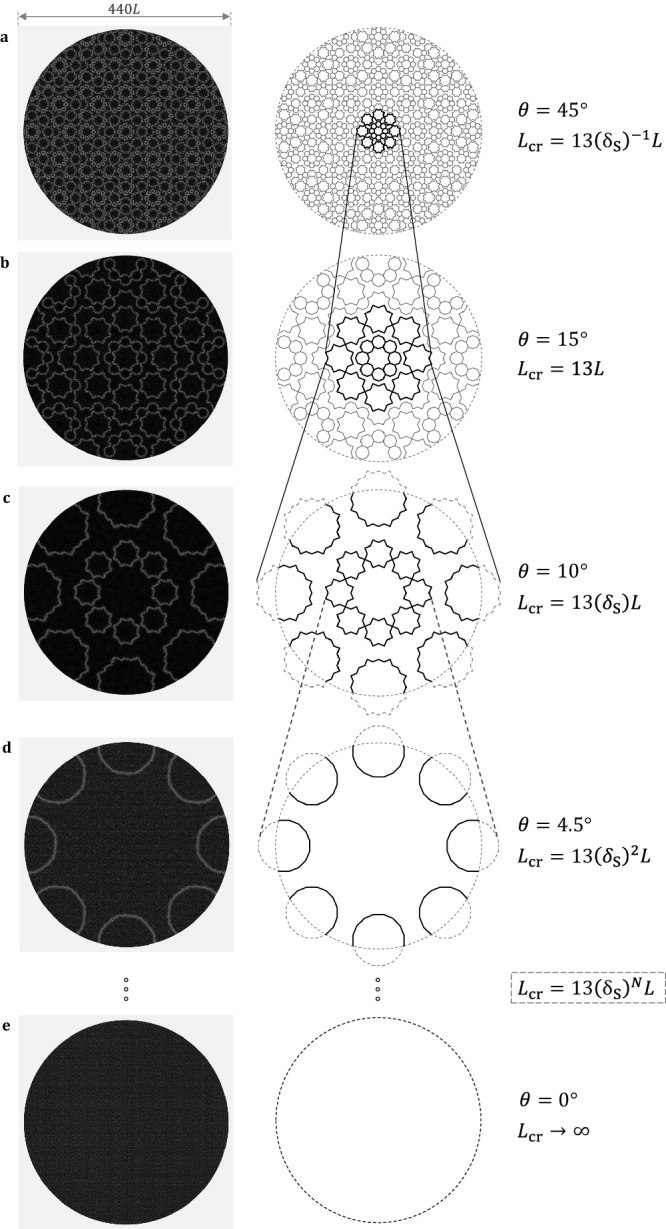
Large-scale pattern formation in samples with decreasing chirality angles. Post-transformation large-scale patterns (simulations) in samples $a$–$e$ with decreasing initial chirality angles (**a**) $\theta=45^\circ$, (**b**) $15^\circ$, (**c**) $10^\circ$, (**d**) $4.5^\circ$, and (**e**) $0^\circ$, corresponding to strain levels and visual deformation amplifications of $\varepsilon=0.0985$($2\times$), $0.0945$($2\times$), $0.074$($2\times$), $0.1$($2\times$), and $0.2$($1\times$), respectively. The identified characteristic lengths ${L}_{{{{\rm{cr}}}}}$ are $13{\left({\delta }_{{{{\rm{S}}}}}\right)}^{-1}L$, $13L$, $13\left({\delta }_{{{{\rm{S}}}}}\right)L$, $13{\left({\delta }_{{{{\rm{S}}}}}\right)}^{2}L$, and “longwave”, respectively, where ${\delta }_{{{{\rm{S}}}}}$ denotes the silver ratio.

The large-scale patterns observed in the weak- and strong-chirality samples suggest a strong correlation between the pattern length scale and the initial chirality; specifically, lower chirality is associated with larger characteristic length scales. To clarify this correlation, we examined additional samples spanning a broader range of microstructural chirality angles. Figure 4 shows the pattern formation for chirality angles $\theta=45^\circ$, $15^\circ$, $10^\circ$, $4.5^\circ$, and $0^\circ$, corresponding to samples $a$–$e$, respectively (see also the animation in Supplementary Movie 4). The corresponding geometric configurations are detailed in Table 1. Note that the configurations $a$ ($\theta=45^\circ$) and $b$ ($\theta=15^\circ$), respectively, correspond directly to the strong ${\chi }_{{{{\rm{S}}}}}$ and weak ${\chi }_{{{{\rm{W}}}}}$ chirality samples reported above. Additional results for configuration $c$ are provided in Supplementary Results 2 and Supplementary Fig. 2. All results were numerically derived from sufficiently large samples with a sample width of ${W}_{{{{\rm{S}}}}}=440L$. Across this set of results, we observed that the highest chirality sample (Fig. 4a, $\theta=45^\circ$) forms the smallest-scale pattern. Reductions in the chirality angle $\theta$ lead to progressively larger-scale patterns, resembling a “magnifying” effect. However, as $\theta$ approaches zero, the growth of the scale appears to extend the observable pattern beyond the finite sample, which we refer to as a “long-wave” mode of pattern formation in this study.

**Table 1:** Geometric parameters and characteristic lengths for samples with various chirality angles

Sample	Chirality	Porosity	Void dimensions			${L}_{{{{\rm{cr}}}}}$
	$\theta$	${\bar{\rho }}_{0}$	$b/L$	$a/L$	$d/L$	
${a}({\chi }_{{{{\rm{S}}}}})$	$45^\circ$	$0.498$	$0.7$	$0.5$	$0.88$	$13{\left({\delta }_{{{{\rm{S}}}}}\right)}^{-1}L$
${b}({\chi }_{{{{\rm{W}}}}})$	$15^\circ$	$0.438$	$0.73$	$0.46$	$0.8$	$13L$
$c$	$10^\circ$	$0.438$	$0.73$	$0.46$	$0.8$	$13\left({\delta }_{{{{\rm{S}}}}}\right)L$
$d$	$4.5^\circ$	$0.431$	$0.717$	$0.451$	$0.8$	$13{\left({\delta }_{{{{\rm{S}}}}}\right)}^{2}L$
$e$	$0^\circ$	$0.438$	$0.73$	$0.46$	$0.8$	${{{\rm{Longwave}}}}$

Columns list the sample label $a$–$e$, chirality angle $\theta$, initial porosity ${\bar{\rho }}_{0}$, void dimensions ($b/L$, $a/L$ and $d/L$), and the resulting characteristic length ${L}_{{{{\rm{cr}}}}}$ of the large-scale pattern, normalized by the prototile side length $L$. The configurations $a$ and $b$ correspond to the strong (Fig. 3) and weak (Fig. 2) chirality samples reported above, respectively. With decreasing chirality angle, the characteristic lengths follow a discrete sequence governed by the silver ratio ${\delta }_{{{{\rm{S}}}}}=1+\sqrt{2}$. The corresponding patterns are shown in Fig. 4.

However, this correlation has so far been observed only at the morphological level, thereby raising a question: can this correlation be quantified geometrically? Our further examination reveals that each large-scale pattern admits a well-defined mapping onto a quasiperiodic “characteristic frame,” as shown in Fig. 4 next to the deformed samples. These frames are not arbitrary; instead, they follow specific “pathways” mapped on the material’s initial Ammann-Beenker tiling, as detailed in the Methods. The characteristic length ${L}_{{{{\rm{cr}}}}}$ of each pattern can then be identified by the prototile size of the corresponding characteristic frame. The procedure for identifying the characteristic length of the large-scale patterns is detailed in the Methods section. As shown in Fig. 4, the characteristic lengths of patterns $a-e$ are identified as ${L}_{{{{\rm{cr}}}}}=13{\left({\delta }_{{{{\rm{S}}}}}\right)}^{-1}L$, $13L$, $13\left({\delta }_{{{{\rm{S}}}}}\right)L$, $13{\left({\delta }_{{{{\rm{S}}}}}\right)}^{2}L$, and a “long-wave” length (${L}_{{{{\rm{cr}}}}}\gg {W}_{{{{\rm{S}}}}}$), where $L$ is the prototile side length in the base tiling and ${\delta }_{{{{\rm{S}}}}}=1+\sqrt{2}$ is the silver ratio.

We find that, as the initial chirality decreases from $\theta=45^\circ$ to $0^\circ$, the magnifying effect of the formed large-scale patterns manifests as a discrete set of increasing characteristic lengths. This set follows a geometric sequence governed by the silver ratio ${\delta }_{{{{\rm{S}}}}}$, expressed by the general relation ${L}_{{{{\rm{cr}}}}}=13{\left({\delta }_{{{{\rm{S}}}}}\right)}^{N}L$, where ${N}=-1$, $0$, $1$, $2$, $\cdots$. This hierarchical scaling originates from geometric constraints in the Ammann–Beenker quasiperiodic tiling on admissible pairs of prototile groupings. In particular, successful pattern formation requires the existence of neighboring prototile groups with (i) identical composition and (ii) eightfold symmetry, whereas such admissible pairs occur only at discrete separations governed by the silver ratio, as detailed in the Large-scale characterization section.

**Fig. 5:**
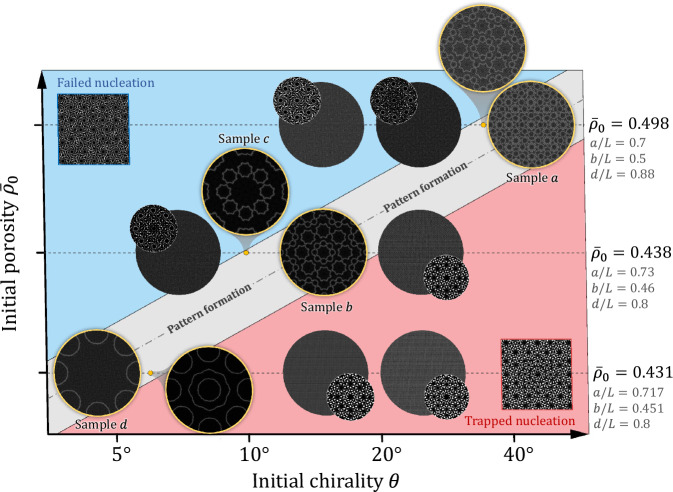
Chirality–porosity parameter space for large-scale pattern formation. The horizontal axis shows the initial chirality angle $\theta$ of the elliptical voids, and the vertical axis shows the initial porosity ${\bar{\rho }}_{0}$. The diagonal gray band denotes a narrow parameter region in which large-scale pattern formation is highly probable, whereas the red and blue regions indicate parameter ranges dominated by failure-to-form mode A (“failed nucleation”) and mode B (“trapped nucleation”), respectively. Source data are provided as a Source Data file.

During our exploration of sample parameters with varying initial chiralities, we find that chirality alone is insufficient to guarantee successful large-scale pattern formation; an appropriate initial porosity is also required. In our configurations, the initial chirality is defined by the chirality rotation $\theta$ of the elliptical voids, while the initial porosity ${\bar{\rho }}_{0}$ is determined by three normalized void dimensions $b/L$, $a/L$, and $d/L$. Accordingly, the geometric design of the material samples can be represented within a two-dimensional parametric space defined by the initial chirality $\theta$ and the initial porosity ${\bar{\rho }}_{0}$. In Fig. 5, we map the outcomes of large-scale pattern formation in this space. The results show that successful pattern formation is restricted to a narrow window defined by a delicate balance between chirality and porosity. Outside this window, the system transitions into two failure-to-form regimes: “failed nucleation”, in which collapse initiates nearly uniformly across the sample without identifiable nucleation, and “trapped nucleation”, in which nucleation initiates but is arrested at small length scales. Specifically, excessively large chirality combined with low porosity leads to “trapped nucleation”, whereas small chirality combined with high porosity promotes “failed nucleation”. The physical origins of this balance between chirality and nucleation behavior are analyzed in the Micro-scale characterization section.

As shown in Fig. 5, for samples with the same initial porosity, there exists a finite window of chirality over which large-scale patterns can form. Increasing porosity systematically shifts this pattern-formation window toward higher chirality. For example, as ${\bar{\rho }}_{0}$ increases from $0.431$ to $0.498$ in Fig. 5, the center of the pattern-formation window shifts from approximately $4.5^\circ$ to $45^\circ$. Within the window at given porosity, lower chirality produces larger characteristic lengths, as illustrated by the samples $c$ and $b$ in Fig. 5. At intermediate chirality angles between samples $c$ and $b$ (Supplementary Fig. 3 and Supplementary Results 3), the characteristic length does not vary continuously but instead falls discretely into one of the two values observed for either sample $c$ or sample $b$. Consequently, accessing larger-scale patterns generally requires shifting the pattern-formation window toward lower chirality, which in turn corresponds to selecting lower initial porosity.

Thus, Fig. 5 serves as a guide for navigating the parameter space to identify large-scale patterns at desired characteristic length scales. However, owing to the strongly nonlinear nature of pattern formation in soft quasiperiodic materials, Fig. 5 should be interpreted as a map of high- and low-likelihood regions, rather than as a strict lookup table.

As observed from both experimental and numerical results, the successful large-scale pattern formation in quasiperiodic porous materials relies on a complete three-stage process: i) localized collapse nucleates at weak spots, ii) propagation of transformation outward from these nascent nuclei, and iii) confinement of the propagation within well-defined domains. These stages are governed by physical mechanisms operating at two distinct scales:

1. Microstructural level (stages i, ii): nucleation and its subsequent breakthrough propagation occur only when the initial geometry satisfies a narrow balance between initial chirality and porosity (Fig. 5). Configurations outside this window, biased toward either excessive chirality or excessive porosity, will evolve into one of two failure-to-form regimes of failed nucleation or trapped nucleation.
2. Large-scale level (stage iii): the final pattern formation requires the advancing transformation fronts to reach a stationary state at specific locations instead of densifying the entire sample, thereby preserving high-porosity pathways.

In short, the formation of large-scale patterns requires the system to avoid a synchronous, spatially uniform transformation across the sample. Instead, scale separation must be established by triggering local instabilities asynchronously at discrete locations. Here, the initial chirality plays a critical role by inducing early-stage softening of elliptical void regions prior to the instability onset, thereby creating asynchrony with the circular void regions that do not experience comparable early softening. This multiscale process is inherently complex and necessitates the successful completion of three consecutive stages, a requirement that explains the rarity of configurations capable of forming large-scale patterns. To physically characterize the three stages, a genuinely multiscale model is required.

In continuum solid mechanics, microstructural transformations in hyperelastic materials are commonly analyzed using the “small-on-large” framework^23^ or finite-amplitude post-transformation analyses^24^. The “small-on-large” method is typically implemented through homogenization or periodic representative volume elements, approaches that rely on translational symmetry and therefore are not directly applicable to quasiperiodic materials. Moreover, homogenization inherently suppresses the asynchronous triggering of local instabilities at discrete locations. Accordingly, in this work, we characterize large-scale pattern formation by resolving distinct length scales: at the microscale, we model the asynchronous softening of the architecture via explicit geometrical characterization; at the large scale, we employ a coarse-grained volumetric analysis to capture the confinement of transformation propagation.

### Micro-scale characterization

**Fig. 6:**
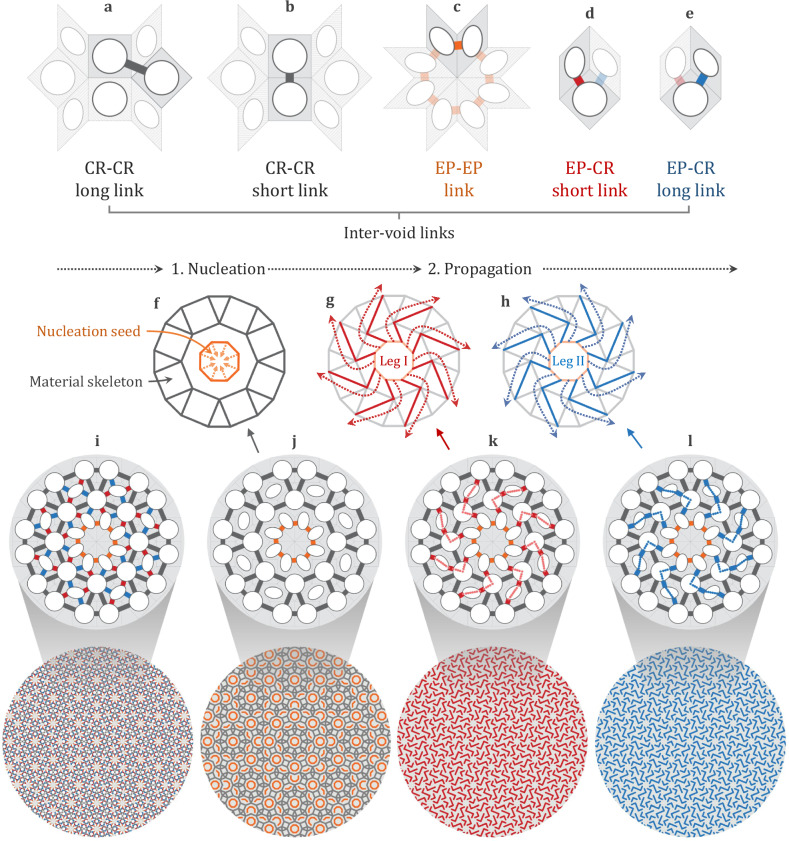
Microstructural model of large-scale pattern formation in soft quasiperiodic materials. Five distinct types of inter-void links in eightfold quasiperiodic porous materials with chiral elliptical voids: Circle–Circle long (**a**) and short (**b**) links, Ellipse–Ellipse (**c**) links, and Ellipse–Circle short (**d**) and long (**e**) links. **i** Schematic illustration of all inter-void links. **f**, **j** Nucleation seeds (array of EE links) trapped within the material skeleton (CC links). Outward propagation of collapse from nucleation seeds through two chiral legs: the primary leg I (**g,** **k**) mediated by Ellipse–Circle short links, and the secondary leg II (**h,** **l**) mediated by Ellipse–Circle long links.

At the small length scale, we construct a microstructural model that elucidates the asynchronous instability mechanism governing the first two stages of pattern formation, namely, the conditions under which nucleation initiates and subsequently propagates rather than being arrested. In this model, the porous architecture is reduced to a network of ligaments connecting neighboring voids, which we refer to as “inter-void” links (Fig. 6). Although the emergent large-scale patterns manifest through void-volume changes (porosity redistribution), the governing micromechanics are carried by these connected ligaments.

In the quasiperiodic porous structures constructed on the Ammann–Beenker tiling with chiral elliptical voids, five distinct types of inter-void links exist, determined by the geometry of adjacent prototiles:

1. Circle–Circle long link (CC–L), Fig. 6a
2. Circle–Circle short link (CC–S), Fig. 6b
3. Ellipse–Ellipse link (EE), Fig. 6c
4. Ellipse–Circle short link (EC–S), Fig. 6d
5. Ellipse–Circle long link (EC–L), Fig. 6e

These links play distinct and complementary roles in the micromechanics of nucleation. Focusing on the vicinity of a nucleation site (Fig. 6f, j), we observe that chiral arrays of EE links form a core that is surrounded by CC links (both CC–L and CC–S). Owing to their chirality, the EE link arrays undergo gradual softening from the beginning of compression, prior to the instability threshold, thereby acting as “nucleation seeds.” By contrast, the CC links assemble into a mechanically intertwined network that is insensitive to chirality and does not exhibit comparable early-stage softening, serving as a “material skeleton” that resists transformation. The resulting asynchronous softening between the nucleation seeds and the material skeleton enables localized instability initiation, namely, the “nucleation” observed in numerical and experimental tests.

The nucleation seeds are initially encapsulated within the skeleton. With continued softening, they reach a critical state at which the skeleton is breached through links bridging the EE arrays to the CC framework, namely, the EC links (Fig. 6k, l). The EC–S and EC–L links act as two mechanical “legs” that enable the nucleation site to pull on and locally rupture the surrounding skeleton, thereby facilitating the outward propagation of collapse. Consequently, the early-stage softening of the nucleation seeds is governed by the effective strength of the EE links, which is modulated by the chirality angle: increasing chirality weakens EE links and thus promotes nucleation. By contrast, the strength of the CC links is controlled solely by porosity: lower porosity produces a stronger CC framework, forming a more robust skeleton that arrests nucleation at an early stage and suppresses further propagation. For the outward propagation legs (EC links), chirality renders the EC–S links significantly shorter and weaker than the EC–L links, thereby establishing a preferential propagation pathway (“chiral leg I,” Fig. 6g). The longer and stronger EC–L links provide a secondary pathway (“chiral leg II,” Fig. 6h). The imbalance between these two legs drives outward propagation along spiral-like trajectories rather than radially. Therefore, stronger chirality increases the curvature of these trajectories, slows propagation, promotes earlier confinement, and results in smaller characteristic lengths.

Based on the above microstructural model, we identify the geometric conditions under which large-scale pattern formation can occur. Across all tested cases, successful configurations consistently satisfy the following ordering of effective inter-void link lengths:

1 $${L}_{{{{\rm{EC}}}}-{{{\rm{L}}}}} > {L}_{{{{\rm{EC}}}}-{{{\rm{S}}}}}\gtrsim {L}_{{{{\rm{EE}}}}}\approx {L}_{{{{\rm{CC}}}}-{{{\rm{S}}}}}$$

where ${L}_{{{{\rm{EE}}}}}$, ${L}_{{{{\rm{CC}}}}}$, ${L}_{{{{\rm{EC}}}}-{{{\rm{S}}}}}$ and ${L}_{{{{\rm{EC}}}}-{{{\rm{L}}}}}$ denote the shortest distances between the corresponding pairs of voids (see the Methods section for details). Configurations that deviate significantly from the relation in Eq. (1) were not observed to develop large-scale patterns in our tests. Examples of the inter-void link lengths are summarized in Supplementary Table 1, where successful pattern formation is observed in samples $a$–$d$, which satisfy the ordering in Eq. (1), whereas samples $\alpha$ and $\beta$ violate this ordering and fail to develop large-scale patterns.

In Eq. (1), the condition ${L}_{{{{\rm{EE}}}}}\approx {L}_{{{{\rm{CC}}}}-{{{\rm{S}}}}}$ indicates that the nucleation seeds and the material skeleton need to possess comparable initial strengths, after which the asynchronous softening of the EE and CC links gives rise to discretely distributed nucleation sites. If ${L}_{{{{\rm{CC}}}}-{{{\rm{S}}}}}$ is too large, the CC links become excessively strong, preventing nucleation from breaking through the material skeleton, and the system consequently enters “trapped nucleation” (sample $\beta$ in Supplementary Table 1 and Supplementary Fig. 1b). Conversely, if ${L}_{{{{\rm{CC}}}}-{{{\rm{S}}}}}$ is much smaller than ${L}_{{EE}}$, the material skeleton is initially too weak to confine the seeds, leading to premature, spatially diffuse collapse rather than localized nucleation. The inequality ${L}_{{{{\rm{EC}}}}-{{{\rm{S}}}}}\,\gtrsim \,{L}_{{{{\rm{EE}}}}}$ ensures that the EE links, acting as nucleation seeds, undergo sufficient early-stage softening before the EC–S links (leg I) collapse, preventing premature propagation. If ${L}_{{{{\rm{EC}}}}-{{{\rm{S}}}}}$ is too small, the propagation legs collapse prematurely, before they can effectively break through the skeleton. However, the condition ${L}_{{{{\rm{EC}}}}-{{{\rm{S}}}}}\gtrsim {L}_{{{{\rm{EE}}}}}$ also imposes a finite upper bound on ${L}_{{{{\rm{EC}}}}-{{{\rm{S}}}}}$. If the EC–S links are excessively longer than the EE links (sample $\alpha$ in Supplementary Fig. 1a and Supplementary Table 1), the corresponding propagation legs become too stiff to collapse, causing them to mechanically trap the EE seeds within the CC framework, rather than facilitating breakthrough of the skeleton. In this case, the seeds fail to evolve into active nucleation sites at the critical strain and the system ends up with a synchronous collapse, leading to the failure-to-form mode “failed nucleation” (Supplementary Fig. 1a). The inequality ${L}_{{{{\rm{EC}}}}-{{{\rm{L}}}}} > {L}_{{{{\rm{EC}}}}-{{{\rm{S}}}}}$ holds whenever the chirality angle is nonzero, but it can also influence the characteristic length of the emergent pattern. The difference $({L}_{{{{\rm{EC}}}}-{{{\rm{L}}}}}-{L}_{{{{\rm{EC}}}}-{{{\rm{S}}}}})$ controls the curvature of the propagation paths: a larger difference (due to stronger chirality) produces more strongly curved, spiral-like propagation, which limits the spatial extent of transformation and results in smaller pattern scales. For example, as shown in Table 1 and Supplementary Table 1, the difference $({L}_{{{{\rm{EC}}}}-{{{\rm{L}}}}}-{L}_{{{{\rm{EC}}}}-{{{\rm{S}}}}})$ increases progressively from sample $d$ to sample $a$, while the characteristic length of the resulting pattern decreases correspondingly from $13{\left({\delta }_{{{{\rm{S}}}}}\right)}^{2}L$ to $13{\left({\delta }_{{{{\rm{S}}}}}\right)}^{-1}L$.

In summary, during stages (i) and (ii) of large-scale pattern formation, nucleation is initiated by seeds formed from arrays of EE links and is either arrested by the CC-link skeleton or propagated outward through EC links. Porosity primarily governs the strength of the skeleton, whereas chirality regulates both the asynchronous softening that enables nucleation and the curvature of the propagation paths. This mechanistic picture is consistent with our experimental and numerical results, which explains why successful pattern formation occurs only within a narrow, balanced range of chirality and porosity (Fig. 5).

### Large-scale characterization

With the microstructural mechanisms governing nucleation and propagation now established, a remaining question is: what mechanism acts to confine the post-breakthrough propagation, preventing it from densifying the entire sample? However, this confinement cannot be captured at the microscale, where the microarchitecture is assumed to collapse once critical compression is reached. Confinement should therefore arise from a large-scale conservation constraint, as further analysis shows that the spatial extent of collapse is limited by the incompressibility of the soft matrix. In fact, the collapse propagation is not a process of eliminating porosity; rather, it is a process of porosity redistribution, which concentrates the remaining porosity into the bright pattern regions.

Under plane-strain equi–biaxial compression, the global porosity of the entire sample is given by

2 $$\bar{\rho }\left(\varepsilon \right)=\,1-\frac{1-{\bar{\rho }}_{0}}{{\left(1-\,\varepsilon \right)}^{2}}\,$$

where ${\bar{\rho }}_{0}$ represents the initial global porosity. In this formulation, the matrix material is assumed to be incompressible, such that the macroscopic volume reduction imposed by compression is accommodated entirely through a reduction of the void volume. Notably, Eq. (2) is independent of any microscale deformation mechanisms. Regardless of how local collapse develops, any local reduction of porosity below the global average must be compensated by porosity increase elsewhere to satisfy global conservation. Equivalently, the total void volume of the sample is determined solely by the applied global strain. After nucleation, the sample contains coexisting collapsed and stable regions with distinct porosity levels, which we refer to as the densified domain ${\Omega }_{{{{\rm{D}}}}}$ and high-porosity domains ${\Omega }_{{{{\rm{P}}}}}$. Equation (2) indicates that the local porosity field must satisfy a global conservation constraint in the undeformed (reference) configuration,

3 $${\int }_{{\Omega }_{{{{\rm{D}}}}}}\rho {dX}dY+{\int }_{{\Omega }_{{{{\rm{P}}}}}}\rho {dX}dY\,=\bar{\rho }\left(\varepsilon \right)\left|{\Omega }_{{{{\rm{S}}}}}\right|$$

where ${\Omega }_{{{{\rm{S}}}}}$ denotes the total area of the sample. Here, $\rho (X,Y)$ denotes the coarse-grained local porosity field defined in the reference configuration. The average porosities in the densified and high-porosity domains are given by

4 $${\bar{\rho }}_{{{{\rm{D}}}}}=\frac{1}{\left|{\Omega }_{{{{\rm{D}}}}}\right|}{\int }_{{\Omega }_{{{{\rm{D}}}}}}\rho {dX}dY,\,{\bar{\rho }}_{P}=\frac{1}{\left|{\Omega }_{{{{\rm{P}}}}}\right|}{\int }_{{\Omega }_{{{{\rm{P}}}}}}\rho {dX}dY$$

Here, we give a quantitative definition of the densified domain ${\Omega }_{{{{\rm{D}}}}}$ as the set of regions with ${\bar{\rho }}_{{{{\rm{D}}}}} < \bar{\rho }$. Accordingly, collapse within ${\Omega }_{{{{\rm{D}}}}}$ does not eliminate porosity; instead, it transfers porosity previously in ${\Omega }_{{{{\rm{D}}}}}$ to the ${\Omega }_{{{{\rm{P}}}}}$ regions during a compensating increase defined by Eq. (3), resulting in ${\bar{\rho }}_{{{{\rm{P}}}}} > \bar{\rho }$. To emphasize this redistribution of porosity, we define the relative porosity field

5 $$\widetilde{\rho }\left(X,Y\right)=\frac{\rho \left(X,Y\right)-\bar{\rho }\left(\varepsilon \right)}{\bar{\rho }\left(\varepsilon \right)}$$

which measures the coarse-grained local porosity relative to the sample average in the reference configuration.

**Fig. 7:**
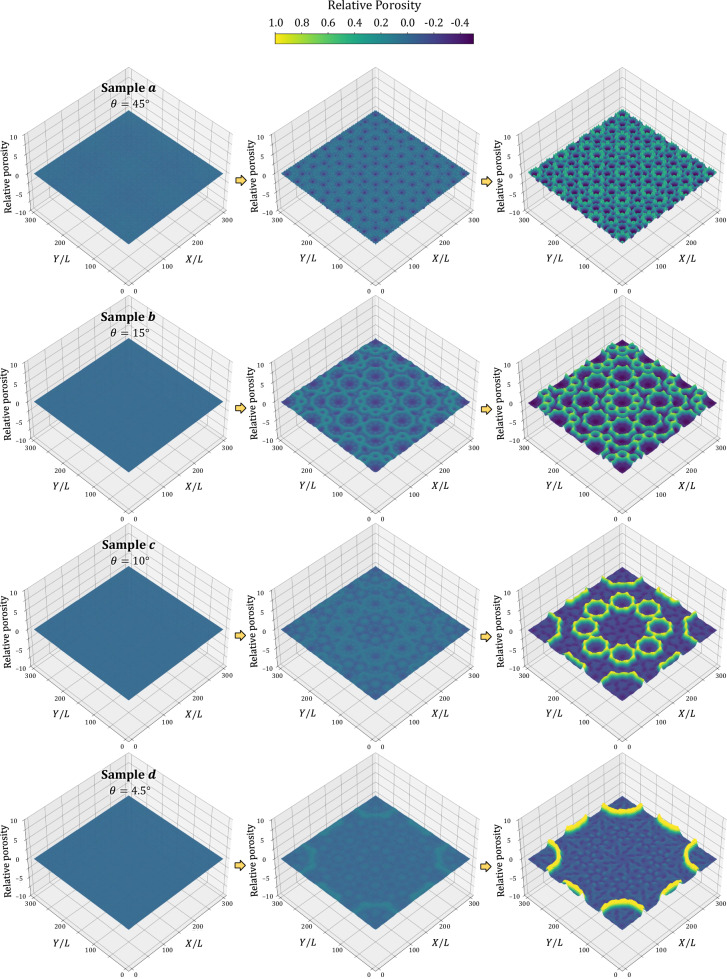
Evolution of the relative porosity field in samples under equi-biaxial compression. **a**–**d** Rows of plots correspond to sample $a$, $b$, $c$, and $d$ with chirality angles $\theta={45}^{\circ }$, $\theta={15}^{\circ }$, $\theta={10}^{\circ }$, and $\theta={4.5}^{\circ }$, respectively. Successive columns, indicated by the yellow arrows, correspond to the undeformed state, the post-nucleation state, and the final pattern-formation state. In each plot, the vertical axis represents the local porosity change relative to the current sample-average porosity. The two horizontal axes correspond to the material-point coordinates defined in the undeformed (reference) configuration. Source data are provided as a Source Data file.

The redistribution of the porosity field can be demonstrated by the evolution of the relative porosity field $\widetilde{\rho }(X,Y)$ during large-scale pattern formation, as shown in Fig. 7 and Supplementary Movie 5. In Fig. 7, each row of plots corresponds to sample $a$, $b$, $c$, and $d$ with chirality angles $\theta={45}^{\circ }$, $\theta={15}^{\circ }$, $\theta={10}^{\circ }$, and $\theta={4.5}^{\circ }$, respectively. The width of each sample is ${W}_{{{{\rm{S}}}}}=300L$, where $L$ is the prototile side length. Successive columns show the field at the (i) undeformed state, (ii) the post-nucleation state, and (iii) the final pattern formation state. We observe that, prior to nucleation, the porosity field is coarse-grained uniform, with local values close to the global average ($\widetilde{\rho }\approx 0$). Once nucleation initiates (see middle column of Fig. 7), the porosity field exhibits a sudden local collapse ($\widetilde{\rho } < 0$) at localized sites, forming densified regions ${\Omega }_{{{{\rm{D}}}}}$ at discrete spots (dark purple troughs), while surrounding regions exhibit a compensating porosity increase above the sample average ($\widetilde{\rho } > 0$), forming high-porosity domains ${\Omega }_{{{{\rm{P}}}}}$ (yellow peaks). At the early post-nucleation stage, high-porosity sites appear in large numbers, far exceeding those that persist after pattern formation (see the last column of Fig. 7). This can be observed during continued compression: the outward propagation of collapse progressively “engulfs” initial high-porosity sites along its path. As formalized in Eq. (3), this engulfment process does not eliminate relative porosity locally but redistributes it, gradually expelling porosity from collapsed regions into the remaining stable regions. Eventually, when multiple propagation fronts encounter one another, the residual porosity is expelled into narrow enough, stripe-like regions (yellow ridges in the last column Fig. 7). Beyond this point, any further propagation would violate the conservation of the total void volume in Eq. (3), which enforces an equilibrium state, preventing further shrinkage of the area ${\Omega }_{{{{\rm{P}}}}}$ and thereby confining propagation and stabilizing the large-scale pattern.

**Fig. 8:**
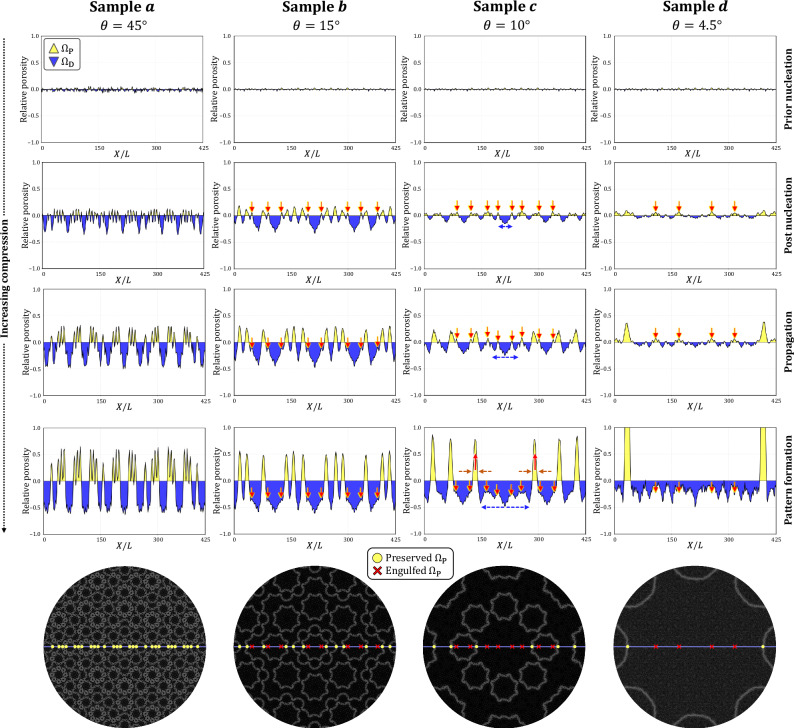
Evolution of the relative porosity field in one-dimensional profiles. The grid of curve plots shows the distribution of relative porosity $\widetilde{\rho }$ along a radial cut of the sample. **a-d** Columns correspond to samples $a$–$d$ with decreasing initial chirality angle $\theta$, and successive rows correspond to increasing levels of compression. Yellow-shaded hills (${\Omega }_{{{{\rm{P}}}}}$) indicate local porosity exceeding the current sample average, whereas blue-shaded valleys (${\Omega }_{{{{\rm{D}}}}}$) denote locally densified domains with porosity below the average. Red arrows mark the locations of initial high-porosity hills that become engulfed by the advancing transformation front. Blue and orange arrows in sample (c) indicate the propagation of transformation. The corresponding large-scale patterns are shown in a separate row and are overlaid with yellow markers denoting preserved ${\Omega }_{{{{\rm{P}}}}}$ hills and red markers indicating ${\Omega }_{{{{\rm{P}}}}}$ hills that are engulfed during the propagation of microstructural collapse. Source data are provided as a  Source Data file.

The propagation of transformation and the evolution of high-porosity regions are more clearly demonstrated by one-dimensional profiles of the relative porosity field $\widetilde{\rho }(X,Y)$, as shown in Fig. 8 and Supplementary Movie 6. The plotted curves represent $\widetilde{\rho }$ evaluated along a radial cut at $Y=150L$ for samples $a$–$d$ shown in Fig. 7, with the horizontal axis denoting the coordinate $X$, normalized by the prototile side length $L$ in the reference configuration. Successive columns correspond to samples $a$–$d$, while the successive rows represent increasing levels of compressive strain.

Figure 8 shows that, before nucleation, the porosity field remains coarse-grained uniform, exhibiting only small, jagged fluctuations originating from the inherent geometric heterogeneity. Once nucleation initiates, localized collapse leads to the formation of densified regions ${\Omega }_{{{{\rm{D}}}}}$ (where $\widetilde{\rho } < 0$), manifested as blue-shaded valleys, as shown in the second row of Fig. 8. Concurrently, compensating high-porosity regions ${\Omega }_{{{{\rm{P}}}}}$ (yellow-shaded hills) simultaneously emerge to maintain porosity conservation imposed by Eq. (3). With further compression, the evolution of ${\Omega }_{{{{\rm{D}}}}}$ and ${\Omega }_{{{{\rm{P}}}}}$ diverges markedly among different samples. In sample $a$, the number of ${\Omega }_{{{{\rm{D}}}}}$ valleys and ${\Omega }_{{{{\rm{P}}}}}$ hills remains essentially unchanged, with both evolving predominantly through increases in amplitude. As a result, the initially formed high-porosity hills survive throughout deformation in sample $a$, giving rise to patterns with relatively small characteristic lengths (Fig. 8a). In contrast, in samples $b$–$d$, certain ${\Omega }_{{{{\rm{D}}}}}$ valleys significantly broaden in area and propagate into neighboring ${\Omega }_{{{{\rm{P}}}}}$ hills (indicated by blue arrows in Fig. 8c). These high-porosity hills are progressively dragged below the sample-average level ($\widetilde{\rho }=0$) and merge into the densified domains (indicated by red arrows in Fig. 8c). However, propagation does not eliminate all ${\Omega }_{{{{\rm{P}}}}}$ hills but instead drives porosity from engulfed regions into the remaining hills. As advancing ${\Omega }_{{{{\rm{D}}}}}$ valleys encounter one another, the intervening ${\Omega }_{{{{\rm{P}}}}}$ hills are compressed, leading to a pronounced increase in amplitude (indicated by orange arrows in Fig. 8c). Ultimately, competing propagation fronts reach equilibrium, thereby confining further propagation and stabilizing pattern formation, as seen in the final row of Fig. 8. From sample $a$ to sample $d$, the number of preserved ${\Omega }_{{{{\rm{P}}}}}$ hills decrease, accompanied by the formation of patterns with progressively larger characteristic length scales. This analysis indicates that the characteristic length of the emergent pattern is governed by the competitive survival of high-porosity regions during collapse propagation.

**Fig. 9:**
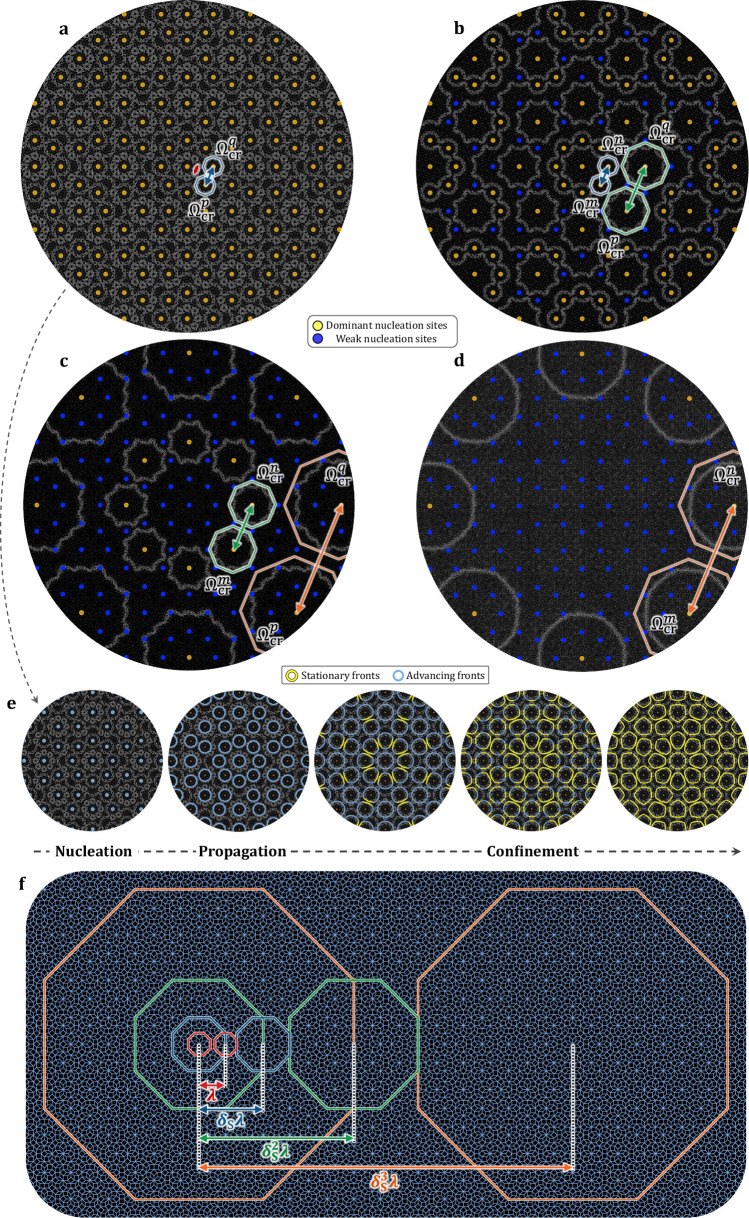
Geometric quantization of hierarchical length scales in large-scale pattern formation. **a**–**d** Final large-scale patterns formed in samples $a$–$d$, respectively. Yellow and blue spots indicate dominant and weak nucleation sites, $\left\{{{{{\boldsymbol{\eta }}}}}_{{{{\rm{cr}}}}}\right\}$ and $\left\{{{{{\boldsymbol{\eta }}}}}_{{{{\rm{w}}}}}\right\}$, respectively. Colored octagons highlight eightfold characteristic domains originating from dominant nucleation sites. The characteristic separations ${\lambda }_{{{{\rm{cr}}}}}^{{{{\rm{I}}}}}$ and ${\lambda }_{{{{\rm{cr}}}}}^{{{{\rm{II}}}}}$ between neighboring pairs of characteristic domains—$\{{\Omega }_{{{{\rm{cr}}}}}^{p},{\Omega }_{{{{\rm{cr}}}}}^{q}\}$ (farther pair) and $\{{\Omega }_{{{{\rm{cr}}}}}^{m},{\Omega }_{{{{\rm{cr}}}}}^{n}\}$ (closer pair)—are indicated by arrows. **e** Schematic visualization of the transformation initiated from dominant nucleation sites in sample $a$. Advancing transformation fronts (blue curves) emanate from dominant nucleation spots and propagate outward until encountering fronts of comparable strength, where stationary equilibrium boundaries form (yellow curves), resulting in confined patterns. **f** Geometric quantization of self-similar neighboring tile-group pairs in the Ammann–Beenker tiling. Each admissible pair consists of domains with identical prototile composition and sufficiently close boundaries to stabilize front interaction. These pairs occur at separations $\lambda$, ${\delta }_{{{{\rm{S}}}}}\lambda$, ${({\delta }_{{{{\rm{S}}}}})}^{2}\lambda$, and ${({\delta }_{{{{\rm{S}}}}})}^{3}\lambda$, forming a geometric sequence governed by the silver ratio ${\delta }_{{{{\rm{S}}}}}$.

The large-scale modeling also facilitates a quantitative interpretation of the observed discrete sequence of characteristic lengths governed by the silver ratio (Fig. 4). As discussed above, the formation of large-scale patterns involves interactions between propagation fronts and the merging of densified domains. Interestingly, in the final patterns of samples $a$–$d$, a substantial number of densified domains remain isolated throughout propagation, allowing them to independently evolve into complete eightfold domains (highlighted by colored octagons in Fig. 9a–d). These local eightfold patterns form the characteristic structural units of the pattern’s characteristic frame (Fig. 4) and are therefore referred to as the “characteristic domains,” denoted by $\left\{{\Omega }_{{{{\rm{cr}}}}}\right\}$. Each characteristic domain evolves from a so-called “dominant nucleation site” $\left\{{{{{\boldsymbol{\eta }}}}}_{{{{\rm{cr}}}}}(X,Y)\right\}$ located at its center (yellow spots in Fig. 9a-d). In contrast, nucleation spots with associated transformation interrupted or engulfed by others are classified as “weak nucleation site” $\left\{{{{{\boldsymbol{\eta }}}}}_{{{{\rm{w}}}}}(X,Y)\right\}$ (blue spots in Fig. 9b-d).

The distribution of $\left\{{{{{\boldsymbol{\eta }}}}}_{{{{\rm{w}}}}}\right\}$ and $\left\{{{{{\boldsymbol{\eta }}}}}_{{{{\rm{cr}}}}}\right\}$ in Fig. 9a–d reveals that the length scale of formed patterns is primarily determined by the separation between characteristic domains (namely, the separation between dominant nucleation sites), marked with colored arrows. In Fig. 9e, we illustrate the formation of the characteristic domains using sample $a$ as an example (see also Supplementary Movie 7). The transformation fronts (blue curves) starting from nucleation site $\left\{{{{{\boldsymbol{\eta }}}}}_{{{{\rm{cr}}}}}\right\}$ remain uninterrupted until they encounter fronts from neighboring nucleation sites, at which equilibrium boundaries are established, resulting in eightfold characteristic domains (yellow curves). Similar formation of characteristic domains can also be observed in sample $b-d$, where the mutually confining characteristic domains are identified in neighboring pairs. In sample $a$, only one such pair is observed, identified as $\{{\Omega }_{{{{\rm{cr}}}}}^{p},{\Omega }_{{{{\rm{cr}}}}}^{q}\}$ in Fig. 9a. In samples $b$ and $c$, two distinct pairs of characteristic domains coexist: a closer pair $\{{\Omega }_{{{{\rm{cr}}}}}^{m},{\Omega }_{{{{\rm{cr}}}}}^{n}\}$ and a farther pair $\{{\Omega }_{{{{\rm{cr}}}}}^{p},{\Omega }_{{{{\rm{cr}}}}}^{q}\}$. In sample $d$, only a closer pair is observable, while the farther pair is assumed to be located beyond the finite sample size. The separation between closer and farther pairs of characteristic domains is quantified by the distance between their associated nucleation sites, namely, ${\lambda }_{{{{\rm{cr}}}}}^{{{{\rm{I}}}}}=\parallel {{{{\boldsymbol{\eta }}}}}_{{{{\rm{cr}}}}}^{p}-{{{{\boldsymbol{\eta }}}}}_{{{{\rm{cr}}}}}^{q}\parallel$ and ${\lambda }_{{{{\rm{cr}}}}}^{{{{\rm{II}}}}}=\parallel {{{{\boldsymbol{\eta }}}}}_{{{{\rm{cr}}}}}^{m}-{{{{\boldsymbol{\eta }}}}}_{{{{\rm{cr}}}}}^{n}\parallel$. According to the definition of the characteristic length ${L}_{{{{\rm{cr}}}}}$ introduced in the Methods section, the farther separation length ${\lambda }_{{{{\rm{cr}}}}}^{{{{\rm{I}}}}}$ in each sample can be directly associated with ${L}_{{{{\rm{cr}}}}}$ via ${\lambda }_{{{{\rm{cr}}}}}^{{{{\rm{I}}}}}=(2+\sqrt{2}){L}_{{{{\rm{cr}}}}}$, while the closer separation length follows ${\lambda }_{{{{\rm{cr}}}}}^{{{{\rm{II}}}}}={({\delta }_{{{{\rm{S}}}}})}^{-1}{\lambda }_{{{{\rm{cr}}}}}^{{{{\rm{I}}}}}$. The separation lengths between characteristic domains identified from samples $a$ to $d$ also increase geometrically with silver ratio, which agrees well with the observed geometric increase of characteristic lengths.

The formation of characteristic domains explains why only a discrete set of characteristic lengths are found in all tested cases, as illustrated in Fig. 9f. As discussed earlier, for two characteristic domains to mutually confine transformation fronts and establish a stationary equilibrium, they must satisfy two geometric conditions: (i) their boundaries must be sufficiently close to interact, and (ii) the two domains must contain exactly identical groups of prototiles. If the two conditions are not met, one domain will inevitably invade the other, preventing the formation of complete eightfold characteristic domains. Geometrically, there is only a discrete set of admissible domain pairs in the Ammann–Beenker tiling that can satisfy these two requirements, as shown in Fig. 9f. The smallest admissible pair (red octagons) has a separation length $\lambda=(3+2\sqrt{2})L$, while larger admissible pairs appear at separations ${\delta }_{{{{\rm{S}}}}}\lambda$, ${({\delta }_{{{{\rm{S}}}}})}^{2}\lambda$, and ${({\delta }_{{{{\rm{S}}}}})}^{3}\lambda$. These separations form a geometric sequence governed by the silver ratio ${\delta }_{{{{\rm{S}}}}}$. The characteristic separation ${\lambda }_{{{{\rm{cr}}}}}^{{{{\rm{I}}}}}$ identified in samples $a$–$d$ belongs to the same sequence but occur at a much larger scale.

The recursive scaling of this sequence by the silver ratio directly reflects the self-similar nature of the Ammann–Beenker tiling: if any domain pair $\{{\Omega }_{p},{\Omega }_{q}\}$ contains an identical set of prototiles, then its scaled counterpart ${\delta }_{{{{\rm{S}}}}}\{{\Omega }_{p},{\Omega }_{q}\}$ still contains identical prototile sets. Consequently, because the confinement stage of large-scale pattern formation requires geometric equivalence between neighboring domains, all admissible characteristic domain pairs — and thus all emergent pattern scales — lie on a discrete geometric sequence determined by the silver ratio.

## Discussion

In this study, we experimentally realized the formation of large-scale patterns in soft quasiperiodic materials that initially featured coarse-grained, spatially uniform void distributions. We reveal that this emergence is governed by a multiscale transformation process comprising three consecutive stages: (i) the nucleation of localized collapse that disrupts the original quasiperiodic order at small length scales, (ii) the outward propagation of microstructural transformations from the nascent nuclei across increasing length scales, and (iii) the eventual stabilization of transformation with re-emergence of quasiperiodic order at large scales. The results demonstrate that this multiscale mechanism is highly sensitive to the balance of initial microstructural chirality and porosity. Successful pattern formation occurs at characteristic length scales that fall into a discrete set governed by the silver ratio. While the reported phenomenon is observed in soft quasicrystals with Amman-Beenker symmetry, the identified key mechanisms can potentially be used to explore and design other types of transformative soft quasicrystals.

Remarkably, the observed coexistence of densified and high-porosity regions during transformation is analogous to first-order-like transformations and phase-coexistence phenomena. The future development of a quantitative thermodynamic or energetic framework for soft quasicrystals (accounting for the inherent microstructural mechanics complexity) can be very instrumental and serve as a new lens for understanding this phenomenon.

We note that, despite undergoing extensive and complex microstructural transformations, the material exhibits path-independent behavior and fully recovers its original configuration upon unloading. This suggests that the extraordinary features of soft quasicrystals^25–27^ can be empowered into tunable and switchable systems^5,28–31^, opening a new class of transformative materials with quasiperiodic order propagating spanning multiple length scales. The space of transformative quasiperiodic microstructures can be potentially expanded even further through leveraging the intrinsic viscoelastic behavior of the soft materials; thus, for example, the material loading history may result in drastically different microstructural configurations evolving between distinct states.

## Methods

### Experiments

The experimental samples were fabricated through a layer-by-layer photopolymerization method by a Stratasys Objet260 Connex3 3D printer. In this study, the samples were composed of a FLX9860 digital material mixture^32^ of two base polymers: Agilus30 (FLX935) and Vero Black Plus (RGD875). Plane-strain conditions were enforced by clamping the material sheet between two rigid acrylic plates. To minimize viscoelastic effects inherent to the elastomeric matrix, we applied a constant, slow loading rate of $2.5\times {10}^{-3}{{{{\rm{s}}}}}^{-1}$. To reduce friction at the contact interfaces and facilitate near-ideal in-plane deformation, silicone oil was applied between the material sample and the acrylic plates.

### Numerical modeling

The numerical simulations were implemented and carried out as follows. First, the microstructure geometry is parametrized for the Ammann–Beenker tiling using the “multigrid method”^33^ implemented in Python 3.10; the resulting geometry is exported as a standard Drawing Interchange Format (DXF) CAD file. The CAD model is then imported into the built-in Geometry module of COMSOL Multiphysics 6.0, where circular and elliptical voids are embedded into the base tiling by introducing four geometric control parameters: the chirality rotation $\theta$ of the elliptical voids, and three void dimensions, $b/L$, $a/L$, and $d/L$, where $L$ denotes the side length of the square and rhombus prototiles.

Computational implementation is carried out in the Solid Mechanics module, where the constructed square sample (width ${W}_{{{{\rm{S}}}}}$) is subjected to equi-biaxial, plane-strain compression (as illustrated in Fig. 1e). The equilibrium equations are formulated under quasi-static loading assumptions, and therefore, due to the assumed slow loading rate in experiments, we do not include rate dependence or inertia. Each prototile is meshed using two-dimensional structural elements with quadratic Lagrange shape functions, where the maximum element size is set to $0.05L$. This ensured a consistent mesh topology across the parametric studies, minimizing potential numerical deviation due to mesh variation.

The neo-Hookean constitutive model was adopted to describe the mechanical behavior of the soft material. Specifically, the corresponding strain energy density function is

6 $$W=\frac{\mu }{2}\left({I}_{1}-3\right)+\frac{\kappa }{2}{\left(J-1\right)}^{2}$$

A high ratio $\varLambda=\kappa /\mu={10}^{3}$ is assigned to maintain a nearly incompressible material behavior, where $\mu$ is the initial shear modulus, $\kappa$ is the bulk modulus, and ${I}_{1}={{{\rm{tr}}}}{{{\bf{C}}}}$ is the first invariant of the right Cauchy-Green tensor ${{{{\rm{C}}}}={{{\rm{F}}}}}^{{{{\rm{T}}}}}{{{\rm{F}}}}$, where ${{{\rm{F}}}}$ is the deformation gradient.

### Identification of characteristic lengths

In this study, we establish an explicit method to identify the characteristic lengths of the formed large-scale patterns. For each pattern (for example, ${\chi }_{{{{\rm{W}}}}}$ and ${\chi }_{{{{\rm{S}}}}}$ samples in Supplementary Fig. 4a), we use polylines to fit the bright stripe patterns, marked with red or yellow frames in Supplementary Fig. 4b. These polylines are not introduced purely by visual inspection; instead, they are constrained by an explicit mapping onto a standard Ammann–Beenker tiling, shown as blue grids in Supplementary Fig. 4b. The resulting polylines define the “characteristic frame” of each pattern, while the corresponding Ammann–Beenker tiling defines the associated “characteristic grid.” The results in Supplementary Fig. 4b demonstrate clear morphological similarity between ${\chi }_{{{{\rm{W}}}}}$ and ${\chi }_{{{{\rm{S}}}}}$ samples: the red frame in the ${\chi }_{{{{\rm{W}}}}}$ sample coincides with a scaled, inner portion of the red frame in the ${\chi }_{{{{\rm{S}}}}}$ sample (Supplementary Fig. 4b). Consistently, the characteristic grid of the ${\chi }_{{{{\rm{W}}}}}$ sample is a magnified version of that of the ${\chi }_{{{{\rm{S}}}}}$ sample. Therefore, the scaling factor between different pattern length scales can be directly determined by comparing the prototile sizes in their associated characteristic grids.

In Supplementary Fig. 4c, we overlay the rhombic prototiles of the characteristic grids from (i) the undeformed sample (gray tiles), (ii) the ${\chi }_{{{{\rm{W}}}}}$ (blue), and (iii) the ${\chi }_{{{{\rm{S}}}}}$ (yellow) samples. The characteristic length is defined as the side length of the rhombic prototile in the characteristic grid. Accordingly, $L$, ${L}_{{{{\rm{cr}}}}}^{{{{\rm{W}}}}}$ and ${L}_{{{{\rm{cr}}}}}^{{{{\rm{S}}}}}$ denote the characteristic lengths in the undeformed configuration and of the post-pattern-transformation ${\chi }_{{{{\rm{W}}}}}$ and ${\chi }_{{{{\rm{S}}}}}$ samples, respectively. From the results, we find that ${L}_{{{{\rm{cr}}}}}^{{{{\rm{W}}}}}$ and ${L}_{{{{\rm{cr}}}}}^{{{{\rm{S}}}}}$ are related by the silver ratio ${\delta }_{{{{\rm{S}}}}}=1+\sqrt{2}$, namely,

7 $$\frac{{L}_{{{{\rm{cr}}}}}^{{{{\rm{W}}}}}}{{L}_{{{{\rm{cr}}}}}^{{{{\rm{S}}}}}}={\delta }_{{{{\rm{S}}}}}$$

as obtained from the geometric mapping illustrated in Supplementary Fig. 4c. In particular, ${L}_{{{{\rm{cr}}}}}^{{{{\rm{W}}}}}$ is exactly $13$ times the initial characteristic length of the undeformed sample (${L}_{{{{\rm{cr}}}}}^{{{{\rm{W}}}}}/L=12\cos \left(\pi /8\right)+5\sin \left(\pi /8\right)=13$). Using the same identification procedure, we determine the characteristic lengths of samples $a-d$ shown in Fig. 4 and Table 1, where ${L}_{{{{\rm{cr}}}}}$ fits into a general formula ${L}_{{{{\rm{cr}}}}}=13{\left({\delta }_{{{{\rm{S}}}}}\right)}^{N}L$.

To validate the analysis of the pattern similarity and characteristic length ratio, we conducted a Structural Similarity Index (SSIM) analysis^34^ to evaluate the similarity between two pattern images by assessing local pixel correlations. The two pattern images are selected as the same as those shown in Supplementary Fig. 4a. Specifically, the image of the large-scale pattern in the strong chirality sample is enlarged by a ratio of $k$, cropped, and then compared to the original-sized large-scale pattern in the weak chirality sample using SSIM (as shown in Supplementary Fig. 5b, c). This process determines the scale ratio at which the two patterns exhibit the highest similarity. Supplementary Fig. 5d plots the similarity index as a function of the reciprocal enlargement ratio ${k}^{-1}$. We observe a peak similarity in the vicinity of ${k}^{-1}=0.414$, implying an enlargement ratio $k\approx 2.414$ (approximately equal to ${\delta }_{{{{\rm{S}}}}}=1+\sqrt{2}$), which agrees well with the prediction in Eq. (7). The enlarged pattern of ${\chi }_{{{{\rm{S}}}}}$ sample is compared to the original-sized pattern of ${\chi }_{{{{\rm{W}}}}}$ sample as shown in Supplementary Fig. 5b, c. The two patterns show high similarity, differing primarily in the thickness of the high-porosity domains.

### Effective inter-void link lengths

Supplementary Fig. 6a-d illustrates the identification of the effective inter-void link lengths ${L}_{{{{\rm{CC}}}}-{{{\rm{L}}}}}$, ${L}_{{{{\rm{CC}}}}-{{{\rm{S}}}}}$, ${L}_{{{{\rm{EE}}}}}$, ${L}_{{{{\rm{EC}}}}-{{{\rm{S}}}}}$, and ${L}_{{{{\rm{EC}}}}-{{{\rm{L}}}}}$. These quantities are defined as the shortest distances between specific pairs of neighboring voids. We emphasize that these link lengths serve as an effective geometric proxy for inter-void connectivity, rather than a rigorous measure of the mechanical strength of individual links, owing to the complex void geometry and the nonlinear deformation of the surrounding ligaments. Specifically, ${L}_{{{{\rm{CC}}}}-{{{\rm{L}}}}}$ denotes the circle–circle link length in square tiles that share a vertex but no common edge, while ${L}_{{{{\rm{CC}}}}-{{{\rm{S}}}}}$ corresponds to the circle–circle link length in square tiles that share one edge. The effective elliptical–elliptical link length ${L}_{{{{\rm{EE}}}}}$ is measured between elliptical voids in adjacent rhombus tiles with one shared edge. For elliptical–circle links in rhombus tiles with one shared edge, two distinct link lengths are identified due to the chirality of elliptical voids: a longer link ${L}_{{{{\rm{EC}}}}-{{{\rm{L}}}}}$ and a shorter link ${L}_{{{{\rm{EC}}}}-{{{\rm{S}}}}}$.

## Supplementary information

Supplementary Information

Description of Additional Supplementary Files

Supplementary Data 1

Supplementary Data 2

Supplementary Movie 1

Supplementary Movie 2

Supplementary Movie 3

Supplementary Movie 4

Supplementary Movie 5

Supplementary Movie 6

Supplementary Movie 7

Transparent Peer Review file

## Source data

Source Data File

## Data Availability

The data generated in this study are provided in the Source Data file and Supplementary Information. Source data are provided with this paper. The Source Data file contains the data underlying Figs. 2, 3, 5, 7, and 8, as well as Supplementary Figs. 2 and 5, including the simulation stress–strain data (Figs. 2a, 3a, and Supplementary Fig. 2a), geometrical parameters (Fig. 5), post-transformation porosity field data (Figs. 7 and 8), and similarity index data (Supplementary Fig. 5d). All other data supporting the findings of this study are available within the article and its Supplementary Information. Source data are provided with this paper.

## Code availability

No new custom code was developed for this study. The analyses were carried out using the built-in numerical implementation in COMSOL Multiphysics 6.0. The tessellation generation^33^ and similarity analysis^34^ used in this work were carried out following the methods reported in the corresponding references.
