# Supplementary material for: Emergence of large-scale patterns in soft quasicrystals
Source: Nat Commun. 2026 Apr 22;17:5525. doi: 10.1038/s41467-026-71816-y (PMC13287776; doi:10.1038/s41467-026-71816-y)
Supplement: Supplementary file 1 — Supplementary Information [file 41467_2026_71816_MOESM1_ESM.pdf]

Emergence of Large-Scale Patterns in Soft Quasicrystals

Dean Chen[a], Nitesh Arora[b,c], Yuhai Xiang[d], Qi Yao[e], Quan Zhang[f,g],

and Stephan Rudykh[f*]

[a] Department of Mechanical and Aerospace Engineering, University of California, Los Angeles; Los Angeles, CA 90095, United States
[b] School of Chemical and Biomolecular Engineering, Georgia Institute of Technology; Atlanta, GA 30332, United States
[c] Department of Mechanical Engineering, Indian Institute of Technology Jodhpur; Rajasthan, 342030, India
[d] Tokyo Electron America, Inc.; Austin, TX 78741, United States
[e] Department of Mechanical Engineering, University of Wisconsin-Madison; Madison, WI 53706, United States
[f] School of Mathematical and Statistical Sciences, University of Galway; Galway, Ireland
[g] Cavendish Laboratory, Department of Physics, University of Cambridge; Cambridge, CB3 0US UK

* Corresponding author. Email: rudykh@mit.edu (S. Rudykh).

Supplementary Information

Supplementary Results 1: Examples of microstructures that fail to form large-scale patterns

In our study, most configurations, explored across a broad microstructural parameter space, fail to develop large-scale patterns due to incomplete progression through three critical stages. Instead, they evolve into one of two failure-to-form modes: mode A ("failed nucleation") or mode B ("trapped nucleation"), as shown in Fig. 5. Here, we present examples of the two modes in which large-scale patterns do not emerge.

Sample β shown in Supplementary Fig. 1b ($b/L = 0.81$, $a/L = 0.432$, $d/L = 0.75$ and $\theta = 35°$) exemplifies a failure-to-form mode B ("trapped nucleation"), in which nucleation is initiated but quickly arrested by the material skeleton (Fig. 6f, j), preventing further propagation. Specifically, as the compressive strain increases from $\varepsilon = 0.05$ to $\varepsilon = 0.08$, the localized nucleation seeds (arrays of EE links) soften in early stages. However, even at high enough compressive strain levels (up to $\varepsilon = 0.11$), the collapse of EE links remains insufficient to trigger the collapse of the EC links; the corresponding propagation legs become too stiff to collapse, preventing the early softened EE seeds from breaking through the skeleton.

In contrast, some other microstructures fail to form large-scale patterns due to the absence of asynchronous nucleation upon the initiation of void collapse, namely, mode A ("failed nucleation"). Instead, structural collapse is triggered simultaneously across the sample. For

example, sample α shown in Supplementary Fig. 1a ($b/L = 0.6675$, $a/L = 0.5$, $d/L = 0.8$ and $\theta = 5°$) undergoes a transition from stable compression to void collapse between $\varepsilon = 0.05$ and $\varepsilon = 0.11$. However, no concentrated nucleation spots are observed; instead, the transformation initiates uniformly and synchronously across the sample. Consequently, no large-scale patterns are observed in the fully collapsed structure, even at a high enough compressive strain ($\varepsilon = 0.18$).

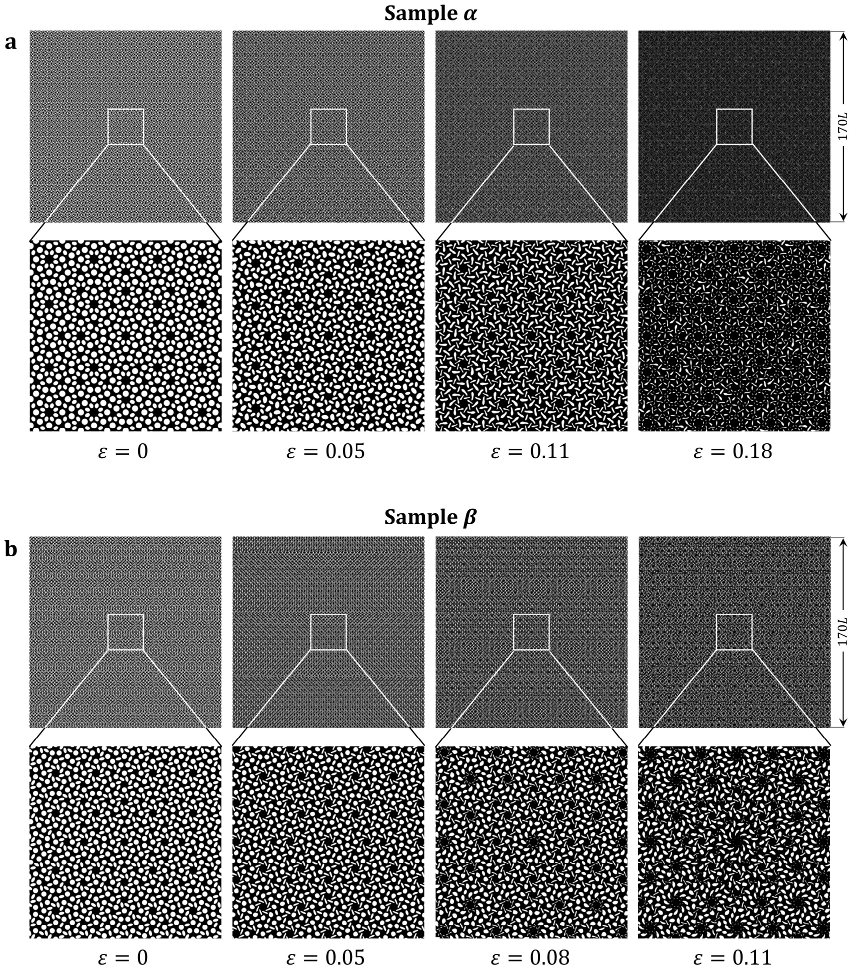

Supplementary Fig. 1 | Examples of microstructures that fail to form large-scale patterns. a Material sample α ($b/L = 0.6675$, $a/L = 0.5$, $d/L = 0.8$ and $\theta = 5°$) at strain levels $\varepsilon = 0$, 0.05, 0.11, and 0.18 (real scale deformation). Void collapse is initiated nearly simultaneously across the sample, resulting in the absence of localized nucleation and thus no pattern formation. **b** Material sample β ($b/L = 0.81$, $a/L = 0.432$, $d/L = 0.75$ and $\theta = 35°$) at strain levels $\varepsilon = 0$, 0.05, 0.08, and 0.11 (real scale deformation).

High chirality leads to excessively weak nucleation seeds, which are not able to break through the material skeleton, thereby preventing further pattern formation.

Supplementary Results 2: Numerical results of large-scale pattern formation in sample c

In this section, we present detailed results for pattern formation in sample c ($b/L = 0.73$, $a/L = 0.46$, $d/L = 0.8$ and $\theta = 10°$), which is modified from sample b (also known as χ_{W} sample, Fig. 2) by reducing the chirality angle to $10°$. Numerical results for this configuration are shown in Supplementary Fig. 2. Specifically, Supplementary Fig. 2a plots the average compressive stress as a function of strain (ε), while Supplementary Fig. 2b and 1c illustrate the corresponding material patterns and their zoomed-in views at various strain levels ($\varepsilon = 0$, 0.03, 0.04, 0.05, 0.055, and 0.0625). We observe that densified domains propagate faster and merge more significantly in sample c compared to sample b (Fig. 2), resulting in an obviously larger scale pattern ($\varepsilon = 0.0625$ in Supplementary Fig. 2b). By simulations on a sufficiently large sample (Fig. 4c), we identify the characteristic length of sample c as $L_{\mathrm{cr}}^{c} = \delta_{\mathrm{S}} L_{\mathrm{cr}}^{b}$, where L_{cr}^{b} is the characteristic length for sample b (χ_{W} sample, Fig. 2). Additionally, compared to sample b, sample c exhibits slightly higher stiffness prior to nucleation, reaches its critical strain earlier, and results in nearly identical stiffness in the post-transformation stage.

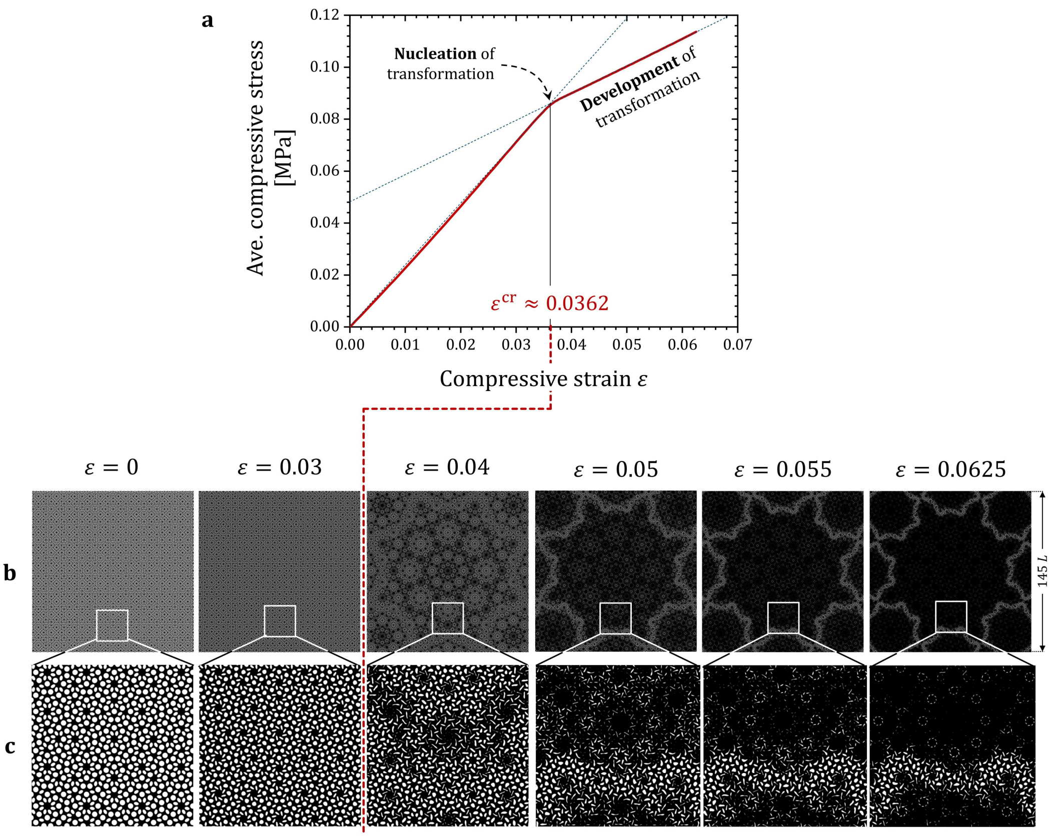

Supplementary Fig. 2 | Numerical results of large-scale pattern formation in sample c ($b/L = 0.73$, $a/L = 0.46$, $d/L = 0.8$ and $\theta = 10°$). a Average compressive stress as a function of equi-biaxial strain. **b** Material sample from prior- to post-transformation states corresponding to the strain level $\varepsilon = 0$, 0.03, 0.04, 0.05, 0.055 and 0.0625 (deformation amplified by 3). **c** Zoom-in images of material samples. Source data are provided as a Source Data file.

Supplementary Results 3: Pattern formation at intermediate chirality angles

To clarify the pattern formation behavior at intermediate chirality angles between samples with identical porosity but different characteristic length scales, we performed a dense chirality sweep over $\theta = 10° - 15°$ for samples with initial porosity $\bar{\rho}_0 = 0.438$ ($b/L = 0.73$, $a/L = 0.46$, $d/L = 0.8$), as shown in Supplementary Fig. 3. As θ increases from 10° (sample c), the characteristic length remains unchanged up to $\theta = 14.5°$. As θ increases from 14.5° to 14.6°, a sharp transition then occurs, with the characteristic length dropping abruptly from $L_{\text{cr}} = 13(1+\sqrt{2})L$ to $L_{\text{cr}} = 13L$. Across all tested configurations, no smooth or continuous evolution of the characteristic length is observed; instead, admissible pattern

scales are restricted to the discrete set $L_{cr} = 13(\delta_S)^N L$, or the system enters a failure-to-form mode.

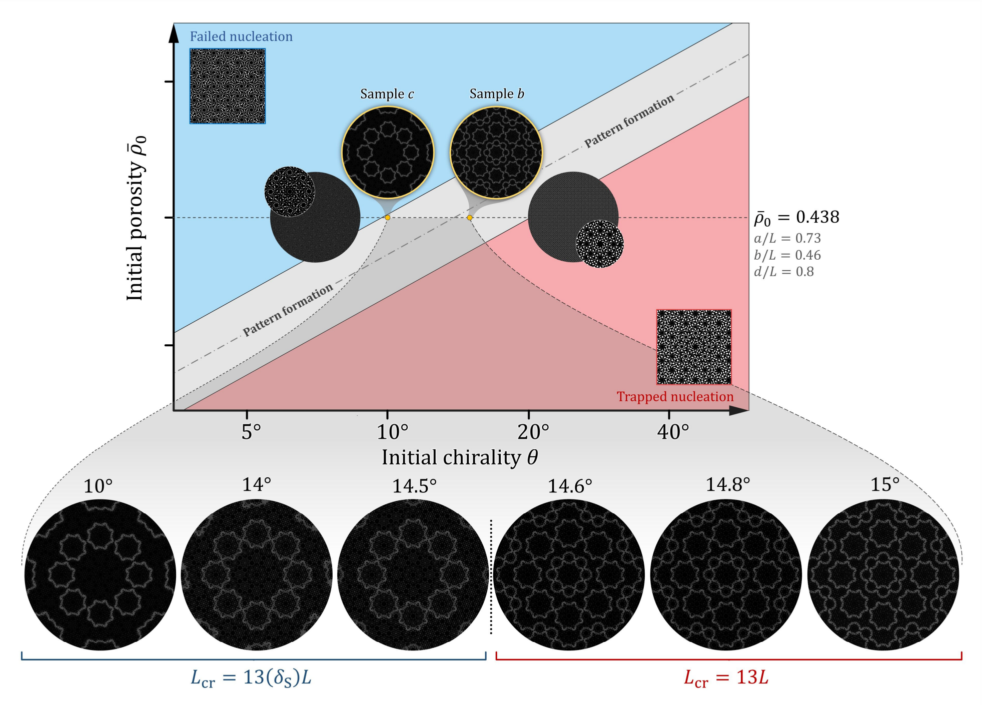

Supplementary Fig. 3 | Example of a refined chirality sweep at fixed porosity across samples with different chirality angles. Dense sweep of the initial chirality $\theta = 10° - 15°$ performed at a fixed initial porosity $\bar{\rho}_0 = 0.438$ ($b/L = 0.73$, $a/L = 0.46$, $d/L = 0.8$), between Sample c and b shown in Fig. 5.

Supplementary Table 1 | Effective lengths of the inter-void links in tested samples. Columns list the sample label a–d, α and β, effective lengths of the inter-void links $L_{\mathrm{EC-L}}$, $L_{\mathrm{EC-S}}$, L_{EE}, and $L_{\mathrm{CC-S}}$. Large-scale pattern formation succeeds in samples $a - d$ and fails in samples α and β.

	**Sample**	$L_{\mathrm{EC-L}}$	$L_{\mathrm{EC-S}}$	L_{EE}	$L_{\mathrm{CC-S}}$
Pattern formation	a (χ_{S})	2.34	1.34	1.17	1.2
	b (χ_{W})	2.62	1.94	1.91	2.0
	c	2.51	2.05	1.95	2.0
	d	2.44	2.23	2.07	2.0
Failure	α	2.4	2.26	1.79	2.0
	β	3.28	1.5	1.5	2.5

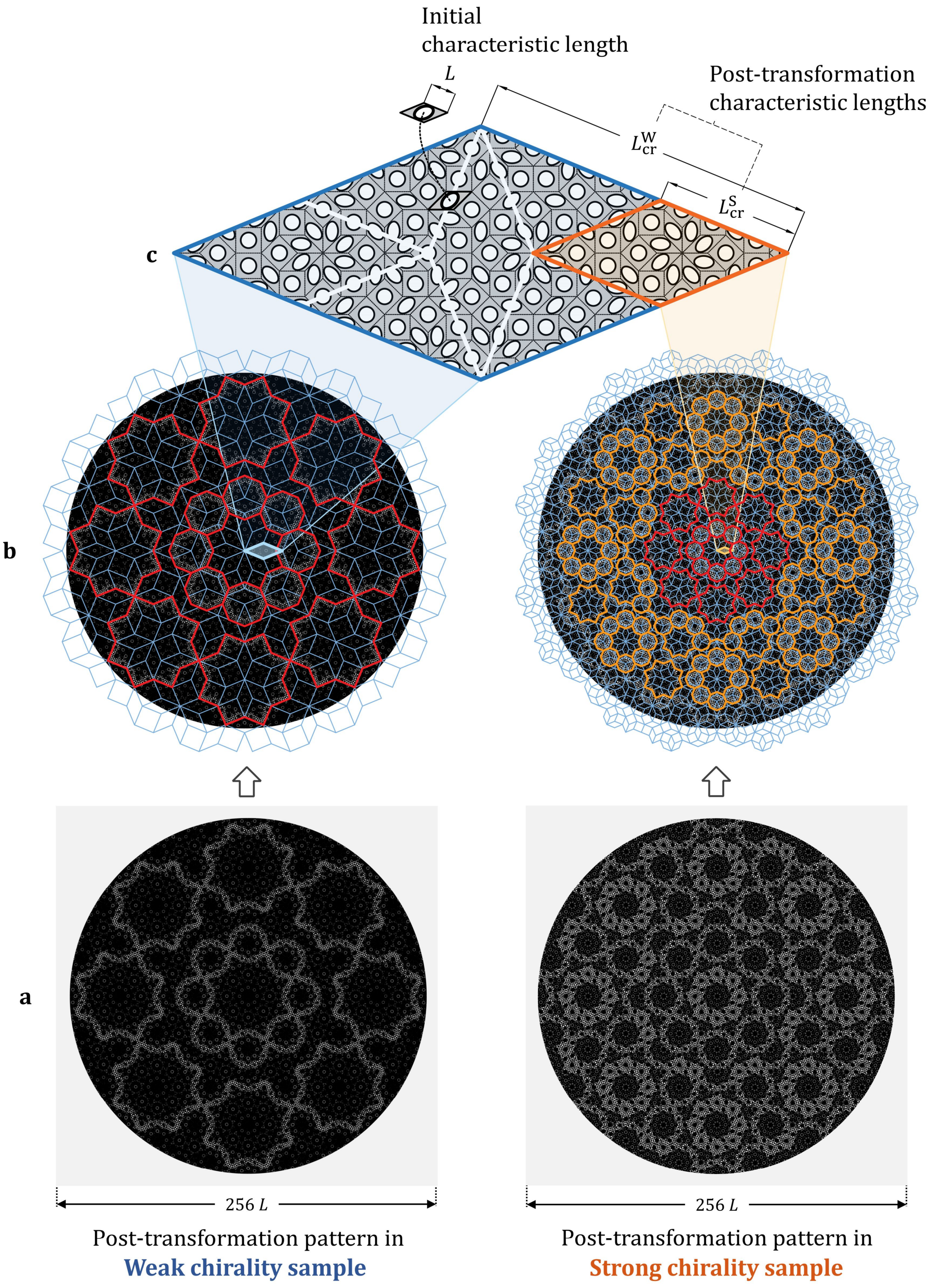

Supplementary Fig. 4 | Identification of characteristic lengths in large-scale patterns. a Large-scale patterns in weak (χ_W) and strong chirality (χ_S) samples from numerical simulations. **b** Mapping of the large-scale patterns (marked by red and yellow polylines) onto characteristic Ammann-Beenker grids (blue grids) in χ_W and χ_S samples. **c** Comparison of characteristic rhombic prototiles in characteristic grids: undeformed (gray), post-transformation samples with weak (χ_W, blue) and strong (χ_S, yellow) chiralities.

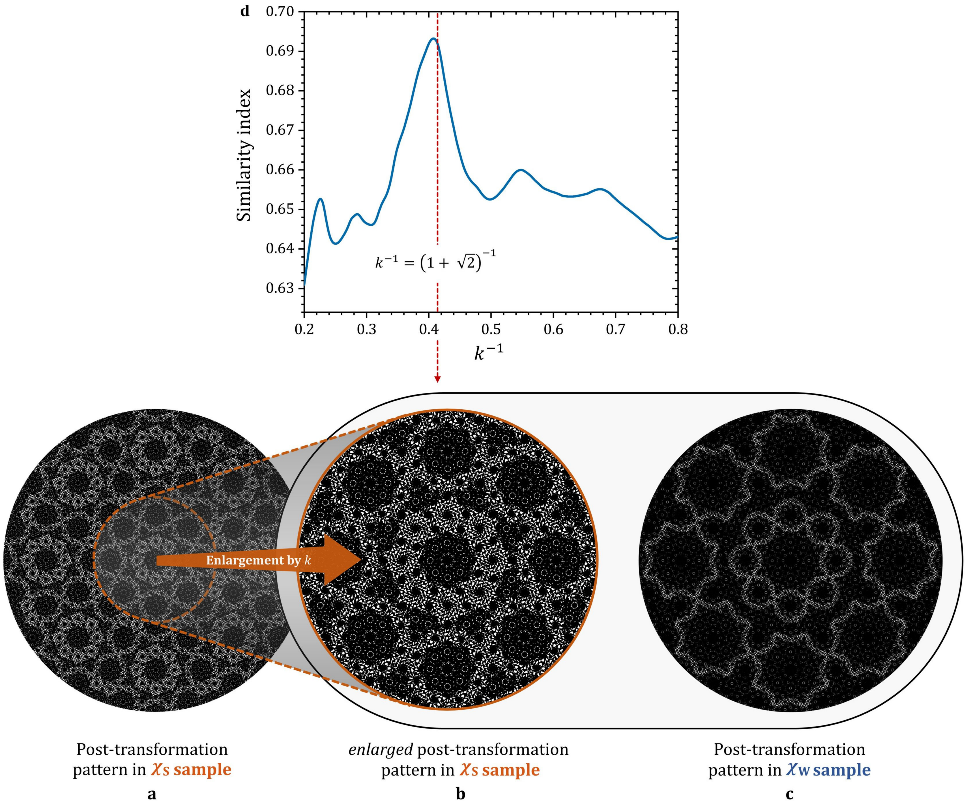

Supplementary Fig. 5 | Structural similarity analysis of large-scale patterns in the weak and strong chirality samples. a Large-scale pattern in strong chirality (χ_S) sample. **b** $1+\sqrt{2}$ enlargement of the large-scale pattern in χ_S sample. **c** Large-scale pattern in weak chirality (χ_W) sample. **d** Similarity index between enlarged χ_S pattern and original-sized χ_W pattern as a function of reciprocal enlargement ratio k^{-1}. Source data are provided as a Source Data file.

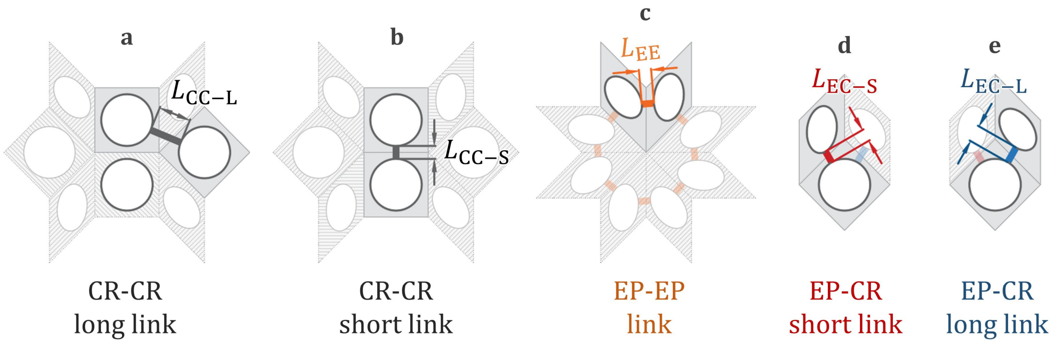

Supplementary Fig. 6 | Identification of effective inter-void link lengths. a–**e** Schematic illustration of the effective inter-void link lengths defined as the shortest distances between neighboring voids in the Ammann–Beenker tiling: Circle–Circle long (**a**) and short (**b**) links, Ellipse–Ellipse (**c**) links, and Ellipse–Circle short (**d**) and long (**e**) links.
