# Supplementary material for: Emergence of large-scale patterns in soft quasicrystals
Source: Nat Commun. 2026 Apr 22;17:5525. doi: 10.1038/s41467-026-71816-y (PMC13287776; doi:10.1038/s41467-026-71816-y)
Supplement: Supplementary file 2 — Description of Additional Supplementary Files [file 41467_2026_71816_MOESM2_ESM.pdf]

Description of Additional Supplementary Files

File Name: Supplementary Data 1

Description: High-resolution photograph of large-scale pattern formation in the weak chirality (χ_{W}) sample at the strain level $\varepsilon = 0.133$.

File Name: Supplementary Data 2

Description: High-resolution photograph of large-scale pattern formation in strong chirality (χ_{S}) sample at the strain level $\varepsilon = 0.135$.

File Name: Supplementary Movie 1

Description: Experimental video of weak chirality (χ_{W}) sample under equi-biaxial plane-strain compression from $\varepsilon = 0$ to $\varepsilon = 0.133$ (video sped up by a factor of 10).

File Name: Supplementary Movie 2

Description: Numerical simulation video of large-scale pattern formation in the weak chirality (χ_{W}) sample from $\varepsilon = 0$ to $\varepsilon = 0.0625$.

File Name: Supplementary Movie 3

Description: Numerical simulation video of large-scale pattern formation in strong chirality (χ_{S}) sample from $\varepsilon = 0$ to $\varepsilon = 0.0985$.

File Name: Supplementary Movie 4

Description: Numerical simulation video of pattern formation in samples a, b, c, and d, with a sample size of $W_{\mathrm{S}} = 440L$.

File Name: Supplementary Movie 5

Description: Evolution of the relative porosity field during equi-biaxial compression in samples a, b, c and d with chirality angles $\theta = 45°$, $\theta = 15°$, $\theta = 10°$, and $\theta = 4.5°$.

File Name: Supplementary Movie 6

Description: Evolution of the relative porosity field in samples a, b, c, and d along one-dimensional profiles at $Y = 150L$. Yellow-shaded hills (Ω_P) indicate local porosity exceeding the current sample average, while blue-shaded valleys (Ω_D) denote locally densified domains with porosity below the average.

File Name: Supplementary Movie 7

Description: Visualization of the transformation initiated from dominant nucleation sites (yellow spots) and its confinement upon encountering fronts from other dominant nucleation sites. Blue curves represent the advancing transformation fronts, and yellow curves denote the stationary equilibrium boundaries.
