# Supplementary figures and images for: Emergence of large-scale patterns in soft quasicrystals
Source: Nat Commun. 2026 Apr 22;17:5525. doi: 10.1038/s41467-026-71816-y (PMC13287776; doi:10.1038/s41467-026-71816-y)

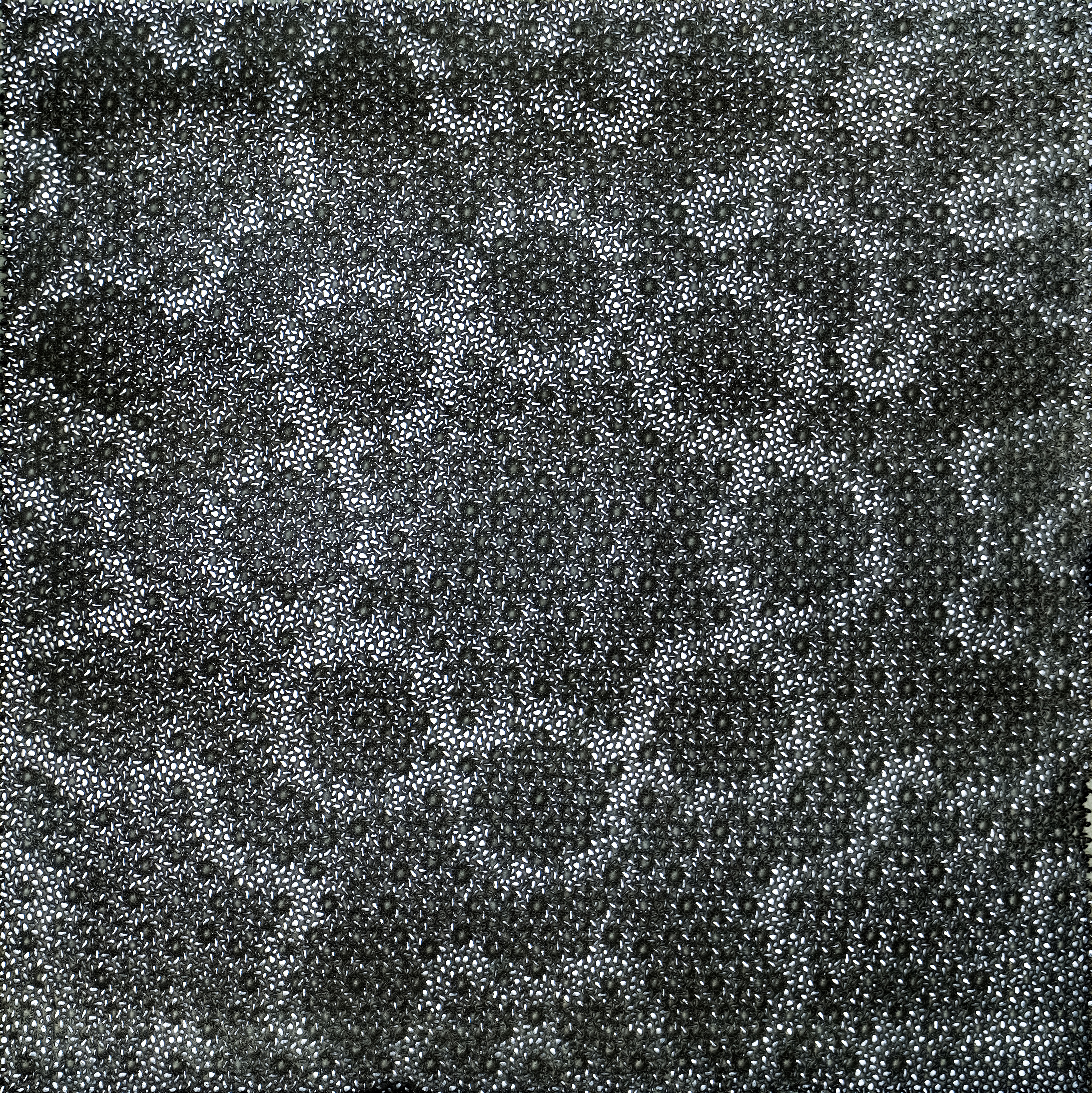

Supplement: Supplementary file 3 — Supplementary Data 1 [file 41467_2026_71816_MOESM3_ESM.jpg]

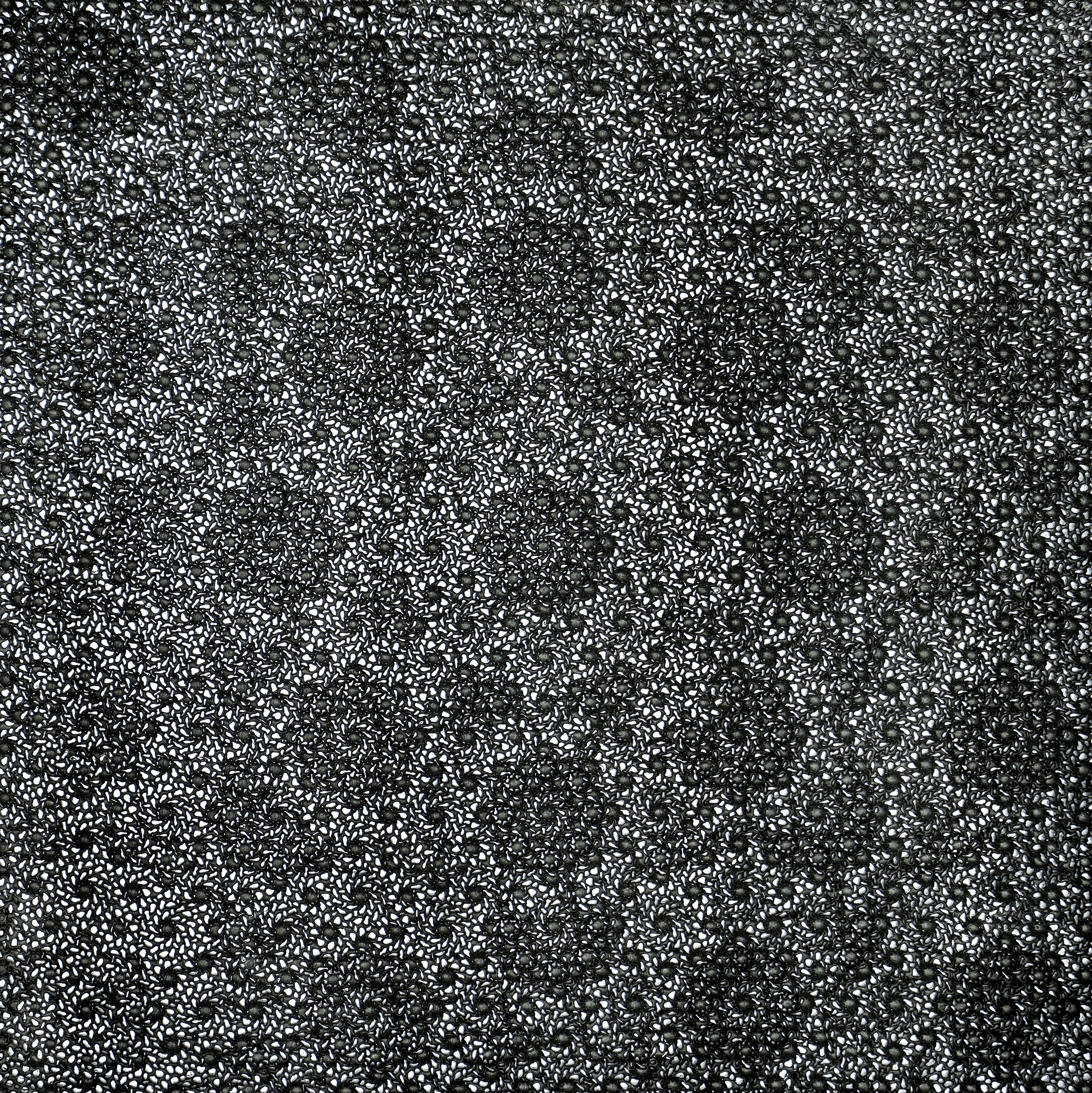

Supplement: Supplementary file 4 — Supplementary Data 2 [file 41467_2026_71816_MOESM4_ESM.jpg]
