# Supplementary material for: Emergence of large-scale patterns in soft quasicrystals
Source: Nat Commun. 2026 Apr 22;17:5525. doi: 10.1038/s41467-026-71816-y (PMC13287776; doi:10.1038/s41467-026-71816-y)
Supplement: Supplementary file 12 — Transparent Peer Review file [file 41467_2026_71816_MOESM12_ESM.pdf]

nature portfolio

Peer Review File

Emergence of Large-scale Patterns in Soft Quasicrystals

Corresponding Author: Professor Stephan Rudykh

This file contains all reviewer reports in order by version, followed by all author rebuttals in order by version.

Version 0:

Reviewer comments:

Reviewer #1

(Remarks to the Author)
In this article the authors explore the patterns that form when a porous material -- whose pore placement is based on a quasicrystal – is deformed under compression. To this end, the authors do both experiment and computer simulations, and show excellent agreement with both. I found this result interesting, and think it would be of interest for the main readership of Nature Communications.

That said, I have a few of points that the authors should address before final publication:

- The paragraph of “Experimental Results” on page 2 and 3 is in a somewhat strange order. I think the authors should reorder the material in this section and start by describing the system they are looking at. (I was somewhat confused until I got to the end of the section).

- There is a typo in the caption of Figure 3 (weak -> strong)

- The description of the simulations is lacking. The authors simply say that "... we performed large deformation analysis using the finite element method (utilizing COMSOL Multiphysics 6.0). " More explanation should be provided: what went into COMSOL, what was the model, parameters, etc.

Reviewer #2

(Remarks to the Author)
The authors describe experiments and numerical results of pattern formation in the deformation of an elastic medium designed with quasiperiodic voids given by a Ammann-Beenker tiling. By adjusting microscopic design parameters such as void ellipticity, orientation (or chirality), and spacing, they note systems in which a larger-scale quasicrystalline pattern emerges in the voids that collapse under strain loading. The results are interesting, but the characterization is incomplete. There are no notable theoretical efforts, and the phenomenon is described at only a few ad-hoc parameter combinations, which limits the potential for future work to build on the manuscript. I can’t recommend publication of the current manuscript unless a significant theoretical characterization is incorporated. Specifically:
1. The phenomenon should be contextualized in terms of the widely known existing thermodynamic theory. Is this modeled well as a first-order equilibrium phase transition? It seems like a phase-coexistence phenomenon influenced by the quasiperiodic medium in which it occurs. Can we understand the critical stress/strain and subsequent softening in terms of a free energy and the Maxwell construction? Can a balance between surface and bulk free energies account for the purported nucleation dynamics?
2. More effort should be made to more exhaustively map out the various parameter regimes than is currently represented in Extended Data Table 1. While there are a large number of free parameters in the lattice design, it would be helpful to constrain all but two and produce a phase diagram showing where the patterns of interest emerge and where they do not (and furthermore, describe what happens for parameters where the quasiperiodic patterns do not emerge). It is especially important to detail whether the emerging patterns (and their purported silver ratio scaling factors) vary continuously with parameters or only at sharp transition points, and whether different patterns with distinct scales can coexist.
3. More microscopic detail on the mechanical instabilities leading to the core collapse could be helpful. Can you characterize a subcritical bifurcation of mechanical equilibria leading to the instability?
Other points:
1. P. 6, on relaxation, does the system return to undeformed state at the same critical strain, or is there some observed

hysteresis?
2. P. 7, What is meant by deformation amplification?
3. P. 9, Fig. 2 (and Fig. 3), please comment more on the differences in the reported strain between the numerical experiments?
4. P. 12, detail how Eq. 1 is derived. What is the meaning of the statement “the soft matrix is assumed to be incompressible”? Isn’t it impossible to biaxially compress an incompressible matrix? Does the deformation actually consist of only shear, and if so, how is the relationship between the strain and global porosity determined? Are there cusps around the collapsed cores where this assumption breaks down?
5. P. 12, What is the meaning of “compression is applied with a uniform speed”? A constant rate of strain, d\varepsilon/dt? Doesn’t Eq. (1) give a nonconstant rate of porosity change (d\rho/dt) given a constant d\varepsilon/dt?

Reviewer #3

(Remarks to the Author)
Review of “Emergence of large-scale patterns in soft quasicrystals” by Chen et. Al.
Chen et.al. present experimental and numerical observations of the response of soft quasiperiodic elastic metamaterials to compression. The metamaterial are elastic sheets imprinted with a quasi-periodic pattern of holes, which is derived from the quasi-periodic Ammann–Beenker tiling. Here the square and rhombi tiles of the Ammann-Beenker are replaced with round and elliptical holes, placed at the corresponding tile’s centroids. The authors find that in a certain regime of parameters (mean porosity, radii of the circles and ellipses) and when introducing certain chirality into the pattern (specifically, to the orientation of the ellipses) the structure’s response to external bi-axial compression is highly nonlinear, characterized by the emergence of a larger-scale quasi-periodic spatial pattern of compressed or collapsed and un-compressed domains. This, the authors find, occurs only for certain values of chirality (rotation of the ellipses by 15 and 45 degrees with respect to the corresponding rhombi). Moreover, the emerging compression patterns recreate the local quasiperiodic motifs at a discrete set of larger scales, related by the silver ratio.

The phenomena convincingly observed by the authors is beautiful and intriguing, and, as far as I can see, new. However, I find the presentation and discussion to be frustratingly lacking. The manuscript contains the design(s) and the (experimental and numerical) observations with a rather detailed phenomenological description of the way the large-scale patterns develop from the different local dynamics as compression progresses, or the microscopic differences between the patterns observed for the two different chiralities. In contrast, the manuscript does not attempt to offer any physical explanation or insight regarding the observed phenomenon. Why do larger-scale quasi-periodic compression patterns emerge? Why are their length-scales related by the silver ratio? Why local chirality is needed to excite these long-range collapse patterns? Why only certain values of the chirality work? More generally, is the observed phenomenon a general property of soft quasicrystals, or could it be observed only in certain types, or perhaps only in this specific example? The authors report that not all parameter values produce the desired large-scale patterns, in fact most of them don’t. Is there any way of predicting where in parameter space this will work and where not?

The analysis presented in the discussion section alternated between the very generic (I believe the mathematical parts will hold for any perforated metamaterial) to the very descriptive (which I find not very convincing based on the raw data, i.e. extended figure and movies). Altogether, the manuscript does not attempt to offer any insight into any of the above questions. Without this, I feel what is left is a nice yet anecdotal observation that does not teach us much. As such, I cannot recommend the publication of this manuscript in Nature Comm.

I am sure some of these questions could be addressed and discussed without extensive effort. For example, the Ammann-Beenker tiling is known to be self-similar, where tiles can be grouped to define bigger tiles that repeat the same quasi-periodic patterns at a larger scale related to the original one by the silver ratio. This well documented fact is not mentioned in the manuscript, but I suspect it might be related to the scaling found in the experiment. Taking this a step further, if the Ammann-Beenker tiling is self-similar across scales, perhaps are also it’s soft-spots, those areas who tend to collapse already at small compressions. This is not trivial but perhaps this could explain why the observed large-scale collapse pattern is a (silver ratio) scaled-up version of the local collapse pattern. This might also hint which quasiperiodic tiling might exhibit this behavior. This argument may be wrong, but I hope the authors find a way to point to the physical mechanisms, generality and limitations of their observations.

Additional comments and remarks:

I am intrigued by Extended Data Fig. 1. The emerging patterns seem to indeed be very sensitive to the chirality angle, which has a magnifying-like effect on the resulting large-scale pattern. Only certain angles are shown, and if I understand correctly, intermediate angles fail to produce a long-range quasiperiodic pattern. Why does this occur? Why does tuning local chirality results in a magnification effect in the collapse patterns?

In page 10, the author describes the observed ‘microscopic’ dynamics leading to the emergence of the ‘macroscopic’ collapse pattern. The description includes nucleation spots, propagating collapse pattern or chain reactions, and ‘gridlocks’ that locally halt propagation. I must say that I cannot identify these phenomenological features in the movies or Figure 3c. I am not sure how the picture was obtained or what the evidence for it is. All I see is a gradual, perhaps two-step compression. Can the authors clarify?

The ‘discussion’ section is not really a discussion. Beyond the generic arguments mentioned above, which I believe are not

unique to quasi-periodic metamaterials, it contains additional and important data analysis. This should be part of the main body of the paper, and a discussion should be added, hopefully with some of the missing physical explanations and insights mentioned above.

In general, I encourage the authors to proof-read their manuscript. Many parts, particularly the non-technical ones, are not written well in my opinion.

Line 139: special -> spatial?

Line 333-334 – reversibility is a basic property of all perforated metamaterials, I don't see why this is considered special here.

Version 1:

Reviewer comments:

Reviewer #1

(Remarks to the Author)
The authors have taken my feedback into consideration and made the alterations I suggested. In particular they have reordered it somewhat and added significant new details on the simulation methods. Hence, I now support publication of this manuscript.

Reviewer #2

(Remarks to the Author)
The authors have substantially revised their manuscript, mostly addressing the points raised by the reviewers. I believe the theoretical description is now adequate for an experimental report. I acknowledge that the theoretical modeling is challenging and that standard homogenization approaches will likely fail to explain the emergent patterns. There may be some more developed theoretical approaches in the pattern formation literature on quasicrystals, but they are likely technical and not widely understood. I believe the authors' revisions have clarified several points, significantly improved the presentation, and positioned the manuscript to provide adequate details to enable other researchers to develop on the discoveries, which are interesting. I recommend publication in Nat. Comm.

Reviewer #3

(Remarks to the Author)
The authors have addressed the primary concerns raised in my initial review In particular, the new Analysis section provides a coherent multiscale physical picture (nucleation–propagation–confinement), a clear qualitative rationale for why chirality is required (asynchronous softening/nucleation seeds vs skeleton), and a geometric explanation for the discrete silver-ratio hierarchy of emergent length scales via admissible self-similar "characteristic domain" pairs in the Ammann–Beenker tiling. These move the work beyond anecdotal observations and provide useful, perhaps predictive guidance.

While the scientific grounding is much improved, In my opinion the manuscript remains dense and, in several sections, quite difficult to read. Specifically, the microstructural analysis is technically thorough but remains cumbersome and with overly complex terminology that may be hard for a general readership. I think portions of the Analysis and Discussion sections would benefit from improved clarity. In addition, there are some strange phrasings here and there. For example, in the introduction: "Aperiodic patterns – with intuitive designs of ancient architectural ornaments bridging to rigorous modern mathematics– have intrigued humanity by their mathematical sophistication, offering substantial potential for the development...". Perhaps it is just me, but I find this strange. There are other examples.

Overall, the manuscript is significantly improved, and key result is novel and interesting. I therefore recommend publication. Nevertheless, I suggest the authors perform a final, rigorous edit to improve the readability and conciseness of their manuscript.

Open Access This Peer Review File is licensed under a Creative Commons Attribution 4.0 International License, which permits use, sharing, adaptation, distribution and reproduction in any medium or format, as long as you give appropriate credit to the original author(s) and the source, provide a link to the Creative Commons license, and indicate if changes were made.
In cases where reviewers are anonymous, credit should be given to 'Anonymous Referee' and the source.
The images or other third party material in this Peer Review File are included in the article's Creative Commons license, unless indicated otherwise in a credit line to the material. If material is not included in the article's Creative Commons license and your intended use is not permitted by statutory regulation or exceeds the permitted use, you will need to obtain permission directly from the copyright holder.
To view a copy of this license, visit https://creativecommons.org/licenses/by/4.0/

Response to Reviewers:

We sincerely thank the Reviewers for their valuable time and constructive feedback. We have carefully considered all comments and incorporated the suggested revisions into the manuscript. Major additions and revisions are highlighted in blue in the revised version.

Response to Reviewer 1:

We sincerely thank the Reviewer for the positive and constructive comments on our work. All points raised have been carefully addressed in the revised manuscript. Specifically, we have revised and reorganized the beginning of the *Experimental Results* section to introduce the material system more clearly, corrected the typo identified in the Fig. 3 caption, and expanded the description of the numerical simulations in the *Methods* section to clarify the modeling procedure and ensure transparency and reproducibility. In addition, we have added a physical characterization of the observed pattern-formation process in the new *Analysis* section.

General comment: *In this article the authors explore the patterns that form when a porous material -- whose pore placement is based on a quasicrystal – is deformed under compression. To this end, the authors do both experiment and computer simulations, and show excellent agreement with both. I found this result interesting, and think it would be of interest for the main readership of Nature Communications.*

Response:

We sincerely thank the Reviewer for this positive assessment and for the recognition of the novelty of our findings and their broad relevance to the readership of Nature Communications.

Comment 1: *The paragraph of "Experimental Results" on page 2 and 3 is in a somewhat strange order. I think the authors should reorder the material in this section and start by describing the system they are looking at. (I was somewhat confused until I got to the end of the section).*

Response:

We thank the Reviewer for this helpful suggestion. We have refined the opening of the Experimental Results section so that it now begins by explicitly introducing the material system under study before proceeding to the details of geometry and loading conditions. Please see lines [57–63] on pages [2-3] of the revised manuscript.

__**Comment 2**__: *There is a typo in the caption of Figure 3 (weak -> strong)*

Response:

We thank the Reviewer for pointing this out. The typo in the caption of Fig. 3 has been corrected, replacing "weak" with "strong" chirality (χ_S). Please see line [240] on page [11] in the revised manuscript.

__**Comment 3**__: *The description of the simulations is lacking. The authors simply say that "... we performed large deformation analysis using the finite element method (utilizing COMSOL Multiphysics 6.0). " More explanation should be provided: what went into COMSOL, what was the model, parameters, etc.*

Response:

We thank the Reviewer for this important comment. Following the Reviewer's suggestion, we expanded the description of the numerical simulations to clearly and explicitly document how the analysis is implemented in COMSOL. Specifically, in the Numerical modeling subsection of the *Methods* section (pages [33-34] in the revised manuscript), we now describe in detail how the base geometry is parameterized and constructed, which physics modules are employed, and how the material behavior and model are implemented under a quasi-static formulation. We believe that these additions make the modeling framework explicit and enable reproducibility of the simulations.

Response to Reviewer 2:

We sincerely thank the Reviewer for finding our results interesting and for the thoughtful feedback. The points raised prompted us to perform a significant volume of additional analyses and simulations, which greatly helped us both broaden and deepen the understanding of the new phenomenon. We added these new results and findings, including new figures and tables, and integrated them with a comprehensive multiscale physical characterization. The expanded results are now organized within a clearer physical framework.

We have made corresponding modifications and clarifications to address the Reviewer's specific comments, as detailed below.

General comment: *The authors describe experiments and numerical results of pattern formation in the deformation of an elastic medium designed with quasiperiodic voids given by a Ammann-Beenker tiling. By adjusting microscopic design parameters such as void ellipticity, orientation (or chirality), and spacing, they note systems in which a larger-scale quasicrystalline pattern emerges in the voids that collapse under strain loading. The results are interesting, but the characterization is incomplete. There are no notable theoretical efforts, and the phenomenon is described at only a few ad-hoc parameter combinations, which limits the potential for future work to build on the manuscript. I can't recommend publication of the current manuscript unless a significant theoretical characterization is incorporated.*

Response:

We thank the Reviewer for the positive assessment of the discovered phenomenon and for the careful and critical feedback on the manuscript. The comments provided valuable guidance that helped us strengthen the manuscript and deepen the physical understanding of the results. As a result, the manuscript has been significantly revised: we have added new results and their analyses (pages [15-17]), introduced new figures (Figs. 5-9, Supplementary Fig. 3) and a new table (Table 2), and incorporated a comprehensive multiscale physical characterization of the phenomenon in the new *Analysis* section (pages [17–31]). The corresponding changes are clarified in detail below.

First, we added newly generated results to expand our dataset and organized them into a systematic parameter-space description, mapping the large-scale pattern formation in the space of initial chirality and porosity (Fig. 5), rather than reporting only a limited set of isolated parameter combinations. Second, we added a multiscale physical characterization that explains the pattern-formation process step by step, from the initial microstructure to nucleation at small length scales, to its subsequent propagation, and finally to the large-scale convergence of pattern formation. These characterizations clarify where large-scale patterns are expected to occur and the mechanisms that drive them. The key mechanisms include:

i) Large-scale pattern formation occurs in samples only within a narrow parameter window defined by a balance between the initial porosity and chirality; outside this window, the system transitions to failure-to-form regimes dominated by either "failed nucleation" or "trapped nucleation." Please see page [17] and Fig. 5 in the revised manuscript.

ii) At the microscale, the void geometry controls whether nucleation can initiate and break through the surrounding ligament network to propagate outward, with porosity and chirality playing distinct and coupled roles. Please see pages [18-23] and Fig. 6 in the revised manuscript.

iii) At the large scale, the incompressibility of the matrix imposes a global porosity conservation that drives the competition between transformation fronts of collapse, thereby stabilizing the formed pattern and preventing collapse of the entire sample. Please see pages [23-28] in the revised manuscript.

iv) The admissible confinement boundaries of the pattern formation (the visually observed bright stripes) are geometrically constrained by the Ammann–Beenker architecture, yielding a discrete set of pattern length scales, which explains the observed characteristic lengths following silver-ratio relations as the chirality decreases. Please see pages [29-31] and Fig. 9 in the revised manuscript.

Comment 1: *The phenomenon should be contextualized in terms of the widely known existing thermodynamic theory. Is this modeled well as a first-order equilibrium phase transition? It seems like a phase-coexistence phenomenon influenced by the quasiperiodic medium in which it occurs. Can we understand the critical stress/strain and subsequent softening in terms of a free energy and the Maxwell construction? Can a balance between surface and bulk free energies account for the purported nucleation dynamics?*

Response:

We thank the Reviewer for the insightful comment. We agree that the observed emergence of large-scale patterns from local collapse events can be contextualized in terms of existing thermodynamic theory, including analogies to phase coexistence and first-order-like transitions.

In our experiments and simulations, biaxial compression of the soft quasicrystalline materials leads to spatially separated densified (collapsed) and high-porosity (uncollapsed) domains. This coexistence of distinct domains resembles a phase-coexistence phenomenon. In classical phase-transition theory, such coexistence is generally associated with a regime where the control parameter lies between the preferred values of the two phases, and is often accompanied by pattern formation through nucleation-like events and front propagation (which are also observed in our system). However, it is important to note that the coexistence here does not occur in a homogeneous medium. As rightly pointed out by the Reviewer, the quasiperiodic geometry directly influences the coexistence by introducing a spatially heterogeneous distribution of local stability thresholds. To characterize this coexistence and its geometric origin, we have introduced a microstructural ligament network description (Fig. 6, page [21]) that captures how asynchronous local transformations emerge from the quasiperiodic architecture, thereby enabling the coexistence of densified and high-porosity domains.

We agree that the reported transformation in this quasiperiodic structured material qualitatively suggests first-order-type behavior and may potentially be modeled as a first-order equilibrium phase transition. However, establishing a rigorous equilibrium phase-transition framework would require constructing an explicit free-energy landscape, including material and geometric nonlinearity, the quasiperiodic distribution of structural features, and the coupling between microstructural transformations and global constraints. To the best of our knowledge, such formulations for free energies have not been developed even for simpler classes of transformative

auxetic materials. This is indeed an exciting research direction, and developing such a framework would require substantial theoretical advances and models to address the inherent structural complexity. We hope that our experimental observations of the phenomenon, together with its numerical analysis-based interpretation, provide the basis for such development. This aspect is included in the concluding remarks of the *Discussion*.

The Reviewer also raised the potential of understanding the critical strain and the post-transformation response through the lens of free energy and Maxwell construction. In several periodic porous materials, phase coexistence is accompanied by a near-plateau response, which can be interpreted using Maxwell-type (common-tangent) arguments based on an effective non-convex energy. In contrast, our material system does not exhibit a clear plateau response; rather, we observe a smooth transition characterized by a decrease in incremental stiffness (please see Fig. 2a and 3a). Another aspect is that standard homogenization-based descriptions underlying Maxwell constructions may not be directly applicable, as they do not capture the spatially asynchronous instabilities intrinsic to quasiperiodic architectures. Therefore, while a generalized energetic formulation (for instance, using simulation-derived effective energies) may be possible, a direct Maxwell construction may not be reliably inferred from the present data.

Lastly, we agree that nucleation dynamics can often be rationalized via competition between bulk driving forces and interfacial costs associated with phase boundaries. Consistent with this general picture, we also observe nucleation, propagation, and front interaction between domains, and the emergent stable pattern is governed by the competitive survival of high-porosity regions during collapse propagation. Formulating an interfacial-energy balance based on local dynamics requires specifying an effective interphase thickness. However, in the present system, this effective interphase thickness is not well defined relative to the evolving microstructure, making its energetic contribution strongly dependent on the chosen definition and potentially reducing it to a fitting parameter. Instead, we interpret domain stabilization using a global porosity-conservation mechanism arising from matrix incompressibility (Eq. (3) on page [24]), which also captures the observed behavior.

In the revised manuscript, we have included a discussion that the observed coexistence of densified and high-porosity regions, together with nucleation and front propagation, bears qualitative similarities to first-order–like transformation behavior. We note that the development of a

quantitative thermodynamic or energetic framework for soft quasicrystals is an exciting and important direction that can potentially serve as a new lens for understanding the phenomenon. Please see the newly added remarks on pages [32], lines [658-662], of the concluding section in the revised manuscript.

Comment 2: *More effort should be made to more exhaustively map out the various parameter regimes than is currently represented in Extended Data Table 1. While there are a large number of free parameters in the lattice design, it would be helpful to constrain all but two and produce a phase diagram showing where the patterns of interest emerge and where they do not (and furthermore, describe what happens for parameters where the quasiperiodic patterns do not emerge). It is especially important to detail whether the emerging patterns (and their purported silver ratio scaling factors) vary continuously with parameters or only at sharp transition points, and whether different patterns with distinct scales can coexist.*

Response:

These suggestions were extremely helpful, and we sincerely thank the Reviewer for providing them. Following the comments, we have recast the parameter space in terms of two characteristic parameters, initial *chirality* and initial *porosity*, and constructed a navigation map based on these parameters for large-scale pattern formation (Fig. 5). This map identifies a parameter window in which large-scale pattern formation is possible. Outside this window, all tested samples fail to form large-scale patterns and instead fall into two distinct failure modes.

Specifically, Fig. 5 shows that large-scale patterns form only with a delicate balance between initial chirality and porosity (gray areas). Excessively large chirality combined with low porosity leads to nucleation that initiates but is arrested by the surrounding material skeleton (“trapped nucleation”, red areas). Conversely, small chirality combined with high porosity leads to collapse that initiates nearly uniformly across the sample without localized nucleation (“failed nucleation”, blue areas).

Our tests show that the characteristic length does not vary continuously with parameters; instead, it takes values from a discrete set governed by the silver ratio. To illustrate this discreteness explicitly, we added results from a dense chirality sweep over the range $\theta = 10° - 15°$ for

samples with the same initial porosity $\bar{\rho}_0 = 0.438$ ($b/L = 0.73$, $a/L = 0.46$, $d/L = 0.8$), as shown in Supplementary Fig. 3 (SI, page [5]). As the initial chirality increases from $\theta = 10°$ (sample c), the characteristic length remains unchanged up to $\theta = 14.5°$. As θ increases from $14.5°$ to $14.6°$, a sharp transition is then observed, with the characteristic length dropping abruptly from $L_{\text{cr}} = 13(1 + \sqrt{2})L$ to $L_{\text{cr}} = 13L$. Across all tested configurations, we do not observe any smooth or continuous evolution of the characteristic length; instead, the characteristic lengths are restricted to the discrete set $L_{\text{cr}} = 13(\delta_S)^N L$, or the system fails to form large-scale patterns.

The mechanism underlying this discreteness is detailed in the newly added *Analysis* section of the revised manuscript (pages [29-31]). In particular, the final patterns are organized by pairs of "characteristic domains" (Fig. 9a–d), which correspond, in the undeformed Ammann–Beenker configuration, to pairs of strictly eightfold-symmetric subdomains composed of identical sets of prototiles. The admissible subdomain pairs occur only at discrete separations that follow a silver-ratio hierarchy (Fig. 9f).

<u>**Comment 3**</u>**:** *More microscopic detail on the mechanical instabilities leading to the core collapse could be helpful. Can you characterize a subcritical bifurcation of mechanical equilibria leading to the instability?*

Response:

We appreciate the Reviewer's helpful suggestion to provide additional microscopic details. Following the Reviewer's suggestion, we performed numerical simulations across a broad range of parameters and introduced a new small-length-scale characterization to resolve the onset and early evolution of transformation (pages [18–23] in the revised manuscript). These results are summarized in a newly added Table 2, which consolidates the microscopic characterization obtained from the expanded result set. These microscopic mechanisms are detailed below.

The microscopic transformation does not initiate simultaneously across the specimen and therefore does not result in a global bifurcation. Specifically, instability is triggered asynchronously and discretely at length scales well separated from the original microstructure: collapse initiates at discrete nucleation sites and propagates outward, while substantial regions remain stable even as some other voids have already fully closed. Consistent with this progressive and spatially non-

uniform evolution, the macroscopic stress–strain responses (Fig. 2a and Fig. 3a) exhibit a smooth change in slope under quasi-static loading, rather than a sharp switch to a hyperelastic behavior.

Microscopic observations further indicate that this scale separation is enabled by the introduction of initial chirality. When the initial chirality is zero, early-stage softening develops uniformly, and instability is triggered synchronously across the specimen, leading to an abrupt change in the stress–strain response. With a nonzero chirality, specific subregions – namely, the arrays of elliptical voids (Fig. 6j) – undergo early-stage softening prior to the critical strain of global instability. These early-softened regions, referred to as "nucleation seeds," are distributed over larger length scales. Before the critical strain, the seeds remain trapped within the surrounding network of non-chiral circular voids, which forms the material skeleton (Fig. 6f). At the critical strain, only a subset of nucleation seeds breaks through this skeleton and initiates outward propagation. Consequently, the macroscopic stiffness decreases progressively as the transformation spreads spatially, rather than through a simultaneous, specimen-wide collapse. Propagation is ultimately confined within specific domains, leaving bright, stripe-like high-porosity pathways whose microstructure remains stable (un-collapsed) voids from the outset.

Other points:

OP (1): *P. 6, on relaxation, does the system return to undeformed state at the same critical strain, or is there some observed hysteresis?*

Response:

We thank the Reviewer for this very interesting and insightful idea. An observable hysteresis would require sufficiently strong viscoelasticity activated either by faster loading and strong material viscoelastic behavior (or both, sometimes appropriate to characterize by the Weissenberg number). Here, the experimental realization focused on the core phenomenon, and we employed the strategy that diminishes those effects (through slow loading), and the numerical model is free of the effects. While we did not observe hysteresis under the conditions studied, such behavior could potentially be realized, opening a rich, transformative behavior of the material system. This is an exciting aspect that we plan to explore, starting with a simpler system and building the complexity up from there. We really appreciate this insight from the Reviewer. In response, we

clarified this in the Experimental Results (line [120-122], page [6]) and added a comment on the potential in the concluding remarks (please see lines [667-671], pages [32-33]).

We also include here, for completeness, the information on what went into the model, the experimental design, and their relations to the hysteresis behavior of the system under the loading conditions used in this work.

In our numerical simulations, we employ a hyperelastic model (with the energy-density function and implementation described in the new part of the *Methods* section on page [34], Eq. (6)), which is typically used for modeling slow-rate deformation of soft materials. As a result of the hyperelastic model choice, the numerical simulations do not produce hysteresis, and the structure attains the same configuration at the same strain during both loading and unloading paths. In this numerical setting, the structure returns to its undeformed configuration once the macroscopic loading is fully released. In reality, soft materials are inherently viscoelastic and may accumulate damage, contributing to hysteresis behavior. In our experiments, we applied a constant slow loading rate (as described in lines [80-81], page [3] in the revised manuscript), so that the viscoelastic effects are weakened; we also employed lubrication to reduce friction (as added in the *Methods* section, lines [680-682] on page [33] in the revised manuscript); but some hysteresis was still observed in the experiments. Nevertheless, the resulting patterns agree well with those obtained from the simulations.

<u>OP (2)</u>: *P. 7, What is meant by deformation amplification?*

Response:

We thank the Reviewer for the opportunity to clarify this point, as it was not described clearly in the original version. The "deformation amplification" is used for illustration purposes by the procedure of presenting the displacement field as uniformly scaled to make the deformed configuration more readily visualized. This amplification is purely graphical (a linear scaling of the displayed displacement field) and does not affect the computed deformation, strain, or stress fields in the finite-element analysis. We have added this clarification to the revised manuscript (page [7], lines [166–168]).

OP (3): *P. 9, Fig. 2 (and Fig. 3), please comment more on the differences in the reported strain between the numerical experiments?*

Response:

We thank the Reviewer for this helpful comment and for the opportunity to clarify the origin of the differences in the reported critical strains between the experiments and simulations. The numerical modeling is performed under idealized approximations, while the experiments diverge from these idealizations. For example, perfect plane-strain equibiaxial conditions cannot be fully enforced experimentally, as out-of-plane deformation cannot be completely suppressed by clamping between acrylic plates, allowing some deformation to be accommodated in the out-of-plane direction; there is also the effect of viscoelasticity, which, despite the global slow strain rate, can activate local effects in the regions experiencing higher strain – this may delay the onset of localized nucleation relative to the idealized hyperelastic response. While these factors contribute to a shift in critical strains, they do not alter the pattern-formation mechanism itself, as evidenced by the close agreement between the patterns observed experimentally and numerically (Figs. 2d,e and 3d,e). Corresponding clarifications have been added to the revised manuscript on pages [8-9], lines [197-203].

OP (4): *P. 12, detail how Eq. 1 is derived. What is the meaning of the statement "the soft matrix is assumed to be incompressible"? Isn't it impossible to biaxially compress an incompressible matrix? Does the deformation actually consist of only shear, and if so, how is the relationship between the strain and global porosity determined? Are there cusps around the collapsed cores where this assumption breaks down?*

Response:

We thank the Reviewer for the opportunity to clarify the assumptions underlying Eq. (1) and the incompressibility assumption for the soft matrix. In our model, the material sample is a two-phase system consisting of (i) an incompressible matrix and (ii) voids whose volume can freely change. The incompressibility assumption refers to the matrix phase only; it does not imply that the

specimen (as a whole) is incompressible. Therefore, the overall volume change under biaxial compression is accommodated primarily through the reduction of void volume.

In reality (in experiments), the matrix polymer shows a very small level of compressibility and is not strictly incompressible; however, any volumetric change of the soft matrix is negligible compared with that of voids, and therefore does not significantly affect the porosity–strain relation used here. We have added clarifications in the revised manuscript (page [24], lines [482-484]).

<u>OP (5)</u>: *P. 12, What is the meaning of "compression is applied with a uniform speed"? A constant rate of strain, d\varepsilon/dt? Doesn't Eq. (1) give a nonconstant rate of porosity change (d\rho/dt) given a constant d\varepsilon/dt?*

Response:

We thank the Reviewer for pointing out this issue and appreciate the opportunity to clarify it. To address the potential ambiguity in the original wording, we have removed the framing in terms of a "speed of porosity change" and instead recast the description in terms of the spatial relative porosity field $\tilde{\rho}$ defined in Eq. (5) and illustrated in Fig. 7 and Fig. 8, which quantifies the local porosity's deviation relative to the sample-average at the same global strain level. Please see the revised sentences in lines [496-502] on pages [24-25] in the revised manuscript.

Response to Reviewer 3:

We sincerely thank the Reviewer for the positive evaluation of the novelty and contribution of this work. The constructive and thoughtful feedback has helped us strengthen the manuscript for both general readers and as a foundation for future studies. In response to the Reviewer's comments, we have obtained and systematically organized new results and developed a new characterization framework to interpret them. The major additions include new results (pages [15–17], and Supplementary Results 3), new figures (Figs. 5–8, Supplementary Fig. 3), and a new table (Table 2). As suggested by the Reviewer, we have also added a comprehensive multiscale physical characterization of the reported phenomena in the new *Analysis* section (pages [17–31]). The detailed responses are provided below.

Comment: *The phenomena convincingly observed by the authors is beautiful and intriguing, and, as far as I can see, new. However, I find the presentation and discussion to be frustratingly lacking. The manuscript contains the design(s) and the (experimental and numerical) observations with a rather detailed phenomenological description of the way the large-scale patterns develop from the different local dynamics as compression progresses, or the microscopic differences between the patterns observed for the two different chiralities. In contrast, the manuscript does not attempt to offer any physical explanation or insight regarding the observed phenomenon. Why do larger-scale quasi-periodic compression patterns emerge? Why are their length-scales related by the silver ratio? Why local chirality is needed to excite these long-range collapse patterns? Why only certain values of the chirality work? More generally, is the observed phenomenon a general property of soft quasicrystals, or could it be observed only in certain types, or perhaps only in this specific example? The authors report that not all parameter values produce the desired large-scale patterns, in fact most of them don't. Is there any way of predicting where in parameter space this will work and where not?*

The analysis presented in the discussion section alternated between the very generic (I believe the mathematical parts will hold for any perforated metamaterial) to the very descriptive (which I find not very convincing based on the raw data, i.e. extended figure and movies). Altogether, the

manuscript does not attempt to offer any insight into any of the above questions. Without this, I feel what is left is a nice yet anecdotal observation that does not teach us much. As such, I cannot recommend the publication of this manuscript in Nature Comm.

Response:

We thank the Reviewer for the high evaluation of the novelty and appeal of the reported phenomena. We particularly appreciate the detailed and insightful questions raised in this comment, which motivated a deeper investigation into the underlying physical mechanisms. Guided by these questions, we obtained new results that bridge the gap between the geometric parameters and the emergence of large-scale patterns. These additions provide a comprehensive physical explanation of the phenomena. The detailed characterization is added to the new *Analysis* section on pages [17-31], which is outlined in detail below.

First, the large-scale patterns emerge because instability does not initiate uniformly across the specimen but instead nucleates asynchronously at a sparse set of localized sites, whose spacing is well separated from the base microstructural length scale. This separation is achieved through a three-stage, multiscale process. (i) At the small length scales, the initial chirality creates local structures that undergo early-stage softening prior to the critical strain; these early-softened sites are referred to as "nucleation seeds" (Fig. 6f), which are initially trapped within a stable network of non-chiral structures, referred to as the "material skeleton" (Fig. 6f). Once sufficient softening of a nucleation seed breaks through the surrounding skeleton, the first stage – nucleation – occurs. (ii) Next, the nucleated collapse propagates outward, progressively breaking the skeleton ligaments along the path, namely, the second stage: "propagation of transformation". (iii) Finally, the pattern formation requires that propagation does not collapse the entire specimen but instead becomes confined. This confinement arises when transformation fronts from different nucleation sites meet, interact, and reach a steady state, defining the third stage: confinement of propagation. The resulting stabilizing equilibrium is governed by global porosity conservation imposed by material incompressibility (Eq. (3)). In short, large-scale patterns emerge because chirality introduces localized, early-softening seeds that trigger transformation earlier than other regions, while the subsequent propagation from those seeds reaches a steady state as they encounter each other.

The relationship between the characteristic length scales and the silver ratio originates from the discrete set of admissible subdomain pairs, whose mutual equilibrium confines the propagation.

Each admissible subdomain possesses strict eightfold symmetry and is composed of an identical set of prototiles as its paired counterpart (referred to as "characteristic domains," Fig. 9a–d). During the third stage of pattern formation, collapse propagation becomes confined once advancing transformation fronts encounter those originating from counterpart subdomains. The final propagation distance, therefore, determines the characteristic length scale of the pattern. This distance corresponds directly to the separation between the pair of characteristic domains (Fig. 9a–d). In the Ammann–Beenker tiling, the admissible characteristic domain pairs are geometrically constrained into a silver-ratio hierarchy (Fig. 9f). A detailed geometric construction and explanation are added in the *Analysis* section (pages [29-31]) and illustrated in the newly added Fig. 9.

Third, as discussed above, initial chirality is essential for separating the length scales associated with the initiation of collapse. In particular, it creates localized "nucleation seed" regions (Fig. 6f) in which the local microstructure undergoes early-stage softening prior to the critical strain, thereby introducing asynchronous softening among different regions before the onset of collapse. This asynchronous initiation is necessary to spatially separate collapse events and enable large-scale reorganization. Without local chirality, collapse is triggered nearly simultaneously and uniformly across the sample, resulting in homogeneous deformation without the formation of spatially organized patterns (please see the "failed nucleation" mode in Fig. 5).

Which values of chirality are effective in pattern formation depends on whether it enables the successful completion of the first two stages: nucleation and propagation. In particular, a delicate balance between the initial chirality and porosity is required. Chirality controls the strength of the nucleation seeds, whereas porosity governs the stiffness of the material skeleton (Fig. 6f,j). As shown in Fig. 5, for a given porosity, excessively large chirality leads to rapid arrest of nucleation ("trapped nucleation"), while too small chirality results in near-simultaneous collapse without identifiable nucleation sites ("failed nucleation").

Finally, we appreciate the Reviewer's question about the potential for realizing the phenomenon in other types of soft quasicrystals. We believe that the phenomenon has the potential to be realizable in other soft quasicrystalline systems, and we hope that the insights obtained into the underlying driving mechanisms may be transferable, with appropriate adaptation, to other quasiperiodic material systems and symmetries. In particular, the key mechanisms elucidated in

this work – namely asynchronous nucleation, early-stage softening, and confinement of propagation – provide a foundation for understanding how large-scale patterns can emerge from initially small-length-scale geometries. At the same time, it should be emphasized that even within the specific system reported here, the emergence of large-scale patterns requires a delicate combination of parameters operating through complex mechanisms. We have added a discussion of these aspects in the concluding remarks (page [32], lines [654-657]).

We also improved the presentation of the results in response to the thoughtful concerns raised by the Reviewer. In the revised manuscript, we move beyond generic arguments and largely descriptive observations and instead provide a qualitative, structured discussion of the results. In particular, we discuss the pattern-formation process within a clear multiscale, three-stage framework. We further adopt a new quantification approach to illustrate the pattern-formation process using the relative porosity field, introduce new plots (Figs. 7 and 8), and animate their evolution in Supplementary Movies 5 and 6, moving beyond a reliance on visual inspection of pattern images.

Comment: *I am sure some of these questions could be addressed and discussed without extensive effort. For example, the Ammann-Beenker tiling is known to be self-similar, where tiles can be grouped to define bigger tiles that repeat the same quasi-periodic patterns at a larger scale related to the original one by the silver ratio. This well documented fact is not mentioned in the manuscript, but I suspect it might be related to the scaling found in the experiment. Taking this a step further, if the Ammann-Beenker tiling is self-similar across scales, perhaps are also it's soft-spots, those areas who tend to collapse already at small compressions. This is not trivial but perhaps this could explain why the observed large-scale collapse pattern is a (silver ratio) scaled-up version of the local collapse pattern. This might also hint which quasiperiodic tiling might exhibit this behavior. This argument may be wrong, but I hope the authors find a way to point to the physical mechanisms, generality and limitations of their observations.*

Response:

We thank the Reviewer for this very helpful suggestion regarding the potential use of the self-similarity of the Ammann–Beenker tiling. This suggestion played an important role in our new

analysis, and helped us in the examination of the similarity between the formed patterns and the intrinsic self-similarity of the Ammann–Beenker tiling. The resulting characterization has been added to the revised manuscript (pages [29–31] and Fig. 9).

In particular, the discrete set of pattern scales is formalized through the formation of "characteristic domains" (Fig. 9a–d). Each of these domains originates from a single nucleation site and evolves without interference from other transformation fronts, such that a complete eightfold-symmetric subdomain forms when its transformation front meets that of a neighboring counterpart. Accordingly, admissible pairs of characteristic domains must satisfy two conditions: (1) each domain is a strictly eightfold-symmetric subdomain of the Ammann–Beenker tiling, and (2) the two domains are composed of identical sets of prototiles, which enables mutual confinement of transformation. Under these constraints, only a discrete set of admissible subdomain pairs exists (Fig. 9f), whose separation distances increase according to the silver ratio δ_S. Owing to the self-similar nature of the Ammann–Beenker tiling, if a subdomain pair $\{\Omega_p, \Omega_q\}$ satisfies conditions (1) and (2), its $\delta_S \times$ scaled counterparts $\delta_S\{\Omega_p, \Omega_q\}$ also satisfy these conditions (Fig. 9f). This recursion follows directly from the self-similar nature of the underlying tiling, such that the entire sequence of admissible length scales is uniquely determined by the silver ratio. As a result, the pattern scales are constrained to a geometric sequence governed by the silver ratio, and all observed patterns belong to a single self-similar family.

Additional comments and remarks:

ACR 1: *I am intrigued by Extended Data Fig. 1. The emerging patterns seem to indeed be very sensitive to the chirality angle, which has a magnifying-like effect on the resulting large-scale pattern. Only certain angles are shown, and if I understand correctly, intermediate angles fail to produce a long-range quasiperiodic pattern. Why does this occur? Why does tuning local chirality results in a magnification effect in the collapse patterns?*

Response:

We are thankful to the Reviewer for pointing out this intriguing behavior. Motivated by these interesting questions, we performed a refined chirality sweep at a fixed porosity to clarify the behavior at intermediate angles, and added these results as *Supplementary Results 3* (SI, pages [4-

5]), with corresponding clarification added in the *Numerical Results* section (lines [318–321], page [16]).

As shown in Supplementary Fig. 3 (SI, page [5]), intermediate chirality angles do not suppress large-scale pattern formation when porosity is fixed. For example, configurations with chirality between sample c ($\theta = 10°$) and sample b ($\theta = 15°$) at the same initial porosity also develop large-scale patterns, but their characteristic lengths fall discretely into one of the two values observed for either sample c or sample b, rather than varying continuously. As θ increases from $10°$ (sample c), the characteristic length remains unchanged up to $\theta = 14.5°$. When θ increases from $14.5°$ to $14.6°$, a sharp transition then occurs, with the characteristic length dropping abruptly from $L_{\mathrm{cr}} = 13(1 + \sqrt{2})L$ to $L_{\mathrm{cr}} = 13L$. Across all tested configurations, we do not observe any smooth or continuous evolution of the characteristic length; instead, the characteristic lengths are restricted to the discrete set $L_{\mathrm{cr}} = 13(\delta_S)^N L$, or the system fails to form large-scale patterns. The physical origin of this discreteness arises from the hierarchical distribution of characteristic domains in the Ammann–Beenker tiling (Fig. 9), as shown in the responses above.

Here, chirality controls the curvature of the propagation paths (Fig. 6g,h) and thereby limits how far collapse can propagate. Specifically, higher chirality induces more strongly curved propagation paths, which reduce the effective propagation distance and thus result in smaller characteristic length scales. Propagation does not terminate continuously, but is arrested by the hierarchical arrangement of characteristic domain boundaries, which act as a discrete set of geometric "checkpoints" (Fig. 9f). Lower chirality allows the propagation to pass through more such checkpoints before confinement, leading to pattern formation at larger length scales, resembling a magnification effect.

ACR 2: *In page 10, the author describes the observed 'microscopic' dynamics leading to the emergence of the 'macroscopic' collapse pattern. The description includes nucleation spots, propagating collapse pattern or chain reactions, and 'gridlocks' that locally halt propagation. I must say that I cannot identify these phenomenological features in the movies or Figure 3c. I am not sure how the picture was obtained or what the evidence for it is. All I see is a gradual, perhaps two-step compression. Can the authors clarify?*

Response:

We thank the Reviewer for pointing out this issue. We acknowledge that in the previous version, the evolution of the pattern-formation process was difficult to discern due to its rapid progression and reliance on purely visual illustrations. Motivated by this comment, we introduced a quantitative descriptor of the pattern-formation process – the relative porosity field $\tilde{\rho}(X,Y)$ (Eq. (5), page [25]). Accordingly, new Figs. 7 and 8 have been added to present the quantitative evolution of $\tilde{\rho}(X,Y)$ during compression. In addition, we created new visualizations in Supplementary Movies 5 and 6 to dynamically illustrate the process.

These newly added illustrations directly visualize the microscopic evolution described in the text. For example, Fig. 8 and Supplementary Movie 6 clearly show the three-stage process of nucleation, propagation, and confinement in the $\tilde{\rho}$ field. In the second row of Fig. 8, multiple peaks and valleys emerge from an initially flat surface; the initial blue valleys correspond to locally collapsed porosity fields at nucleation sites. With increasing compression (third row of Fig. 8), these valleys grow both in amplitude (porosity reduction) and width (spatial propagation), progressively engulfing neighboring peaks, corresponding to the propagation of the transformation. As propagation proceeds, the remaining high-porosity regions (yellow peaks) are squeezed and amplified, preventing them from being further engulfed and thereby forming stable high-porosity pathways, which mark the confinement of propagation. The same process can also be observed in the full-field evolution shown in Fig. 7 and Supplementary Movie 5.

In addition, we have revised the terminology in the *Analysis* section to reflect the underlying physical mechanisms. We have removed confusing descriptors such as "gridlocks" and "chain reactions" and instead use the appropriate terms, such as the "material skeleton" (Fig. 6f) and chiral paths of propagation (Fig. 6g, h). These revisions ensure that the described microscopic dynamics are directly supported by quantitative fields and clearly identifiable in the figures and movies.

<u>ACR 3</u>: *The 'discussion' section is not really a discussion. Beyond the generic arguments mentioned above, which I believe are not unique to quasi-periodic metamaterials, it contains additional and important data analysis. This should be part of the main body of the paper, and a*

discussion should be added, hopefully with some of the missing physical explanations and insights mentioned above.

Response:

We thank the Reviewer for this helpful suggestion. In response, we have substantially restructured the manuscript to clearly distinguish among results, analysis, and discussion, as follows:

i) Key results central to the main findings—including material previously presented in Extended Data Fig. 1—have been moved into the *Numerical Results* section (pages [12–15]).

ii) Procedures for identifying characteristic length scales have been relocated from the *Discussion* to the *Methods* section (pages [34–37]).

iii) Key analyses, including newly added physical characterizations, have been consolidated into a dedicated *Analysis* section (pages [17–31]), which directly addresses the Reviewer's questions by explaining why large-scale patterns emerge, the regions of parameter space in which they occur, and the origin of their discrete characteristic length scales.

Following this reorganization, the *Discussion* section (pages [32–33]) now focuses on summarizing the main findings of the study and outlining their broader implications and potential future directions.

ACR 4: *In general, I encourage the authors to proof-read their manuscript. Many parts, particularly the non-technical ones, are not written well in my opinion.*

Response:

We thank the Reviewer for this careful comment. We have thoroughly proofread the entire manuscript and revised the wording throughout to improve clarity, precision, and readability across all sections.

ACR 5: *Line 139: special -> spatial?*

Response:

We thank the Reviewer for pointing out the typo at line 139 (previous draft). The word “special” should indeed be “spatial.” In the revised manuscript, we have carefully checked for and corrected similar typographical errors. In addition, the original sentence around line 139 in the previous draft has been removed, and the observations of three-stage pattern formation in experiments have been revised and relocated to lines [148–153] on page [7] in the revised manuscript.

ACR 6: *Line 333-334 – reversibility is a basic property of all perforated metamaterials, I don’t see why this is considered special here.*

Response:

We thank the Reviewer for this thoughtful comment. Because the material undergoes very complex multiscale microstructural transformations that could result in path-dependent behavior through mechanisms such as local multi-stability, self-locking, or geometric entanglement, we included an explicit check of reversibility. Despite the micromechanical complexity, the material response remains fully reversible and path-independent. We have retained this result and also added clarification in the revised manuscript (lines [663–665], page [32]).
